# Prevalence and Trends of Not Receiving a Dose of DPT-Containing Vaccine Among Children 12–35 Months: An Analysis of 81 Low- And Middle-Income Countries

**DOI:** 10.1007/s44197-024-00294-6

**Published:** 2024-09-19

**Authors:** Omar Karlsson, Sunil Rajpal, Mira Johri, Rockli Kim, SV Subramanian

**Affiliations:** 1 Duke University Population Research Institute, Duke University, 140 Science Dr, Durham, NC 27710 USA; 2 Centre for Economic Demography, School of Economics and Management, Lund University, P.O. Box 7083, Lund, 220 07 Sweden; 3 Department of Economics, FLAME University, Pune, India; 4 Carrefour de l’Innovation, Centre de Recherche du Centre Hospitalier de l Universite, de Montréal (CRCHUM), Montréal, QC Canada; 5 Département de Gestion, d’Évaluation, École de Santé Publique, et de Politique de Santé, Université de Montréal (ÉSPUM), Montréal, QC Canada; 6 Division of Health Policy & Management, College of Health Science, Korea University, 145 Anam-ro, Seongbuk-gu, Seoul, 02841 Republic of Korea; 7 Interdisciplinary Program in Precision Public Health, Department of Public Health Sciences, Graduate School of Korea University, 145 Anam-ro, Seongbuk-gu, Seoul, 02841 Republic of Korea; 8 Harvard Center for Population and Development Studies, 9 Bow Street, Cambridge, MA 02138 USA; 9 Department of Social and Behavioral Sciences, Harvard T.H. Chan School of Public Health, Boston, MA 02115 USA

**Keywords:** DPT-containing vaccines, Zero dose children, Low- and middle-income countries, Average annual change

## Abstract

Not receiving a DPT-containing vaccine in early childhood indicates an absence of routine immunization, which puts children at an elevated risk of mortality, morbidity, and worse human development over the life course. We estimated the percentage of children 12–35 months who did not receive a dose of DPT-containing vaccine (termed zero-dose children) using household surveys from 81 low- and middle-income countries conducted between 2014 and 2023. For 68 countries with more than one survey (with the earlier survey conducted 2000–2013), we estimated the average annual percentage point change in prevalence of zero-dose children between the earliest and latest surveys. We also explored the association of zero-dose prevalence with postneonatal and child mortality, health expenditure, and Gavi-eligibility. Overall, 12% of children in our pooled sample had not received a dose of DPT-containing vaccine. There was a 0.7% point decline in zero-dose prevalence per year on average across the period studied. A single percentage point average annual decline in zero-dose prevalence was associated with an average annual decrease of 1.2 deaths in the postneonatal and childhood period per 1000 live births. Gavi-eligible countries had a much faster decline in zero-dose prevalence than other countries. Large gains have been made in reducing the percentage of children who did not receive a DPT-containing vaccine. Efforts to reduce the number of zero-dose children should focus on countries with high prevalence to achieve the Immunization Agenda 2030. Healthcare spending could be prioritized so that the prevalence of zero-dose children is reduced.

**Supplementary Information:**

The online version contains supplementary material available at 10.1007/s44197-024-00294-6.

## Introduction

Since the launch of the Expanded Program on Immunization in 1974, mass distribution of childhood vaccines has saved millions of lives [1]. For example, DPT-containing vaccines prevent pertussis and tetanus, which can be life-threatening, particularly in young children without fully developed immune systems [2]. In addition to the clinical importance, not receiving the first dose of DPT-containing vaccine in the first year of life further signals lack of access to routine immunizations and is used, for example, by Gavi, the Vaccine Alliance and the WHO’s Immunization Agenda 2030 to approximate children not receiving any routine vaccine, or “zero-dose children” [3, 4].

Zero-dose children are more vulnerable to vaccine-preventable diseases (such as diphtheria, pertussis, tetanus, polio, Haemophilus influenzae type b [Hib], hepatitis B, measles, pneumonia, and rotavirus diarrhea). Zero-dose children simultaneously experience many forms of adversity, such as conflict, low living standards [5, 6], and limited access to health services [4, 7, 8], which places them at higher risk of poor health and nutritional outcomes, stunted growth, and suboptimal human development and health over the life course [9]. The Immunization Agenda 2030 aims to reduce the prevalence of zero-dose children by 50% (from 2020 levels) by 2030 at the country, regional, and global levels [10].

In the past, diphtheria, pertussis, and tetanus were widespread and caused significant morbidity and mortality, but thanks to vaccination, they are now rare [11]. For example, these diseases killed an estimated 160 thousand children 1–4 years old in 1990, which was reduced to 60 thousand in 2019 [12]. DPT-containing vaccines are recommended for all children, with the primary series of three doses usually given at 6, 12, and 14 weeks of age [13]. (A booster dose of DPT-containing vaccine is recommended at 12–23 months, with additional boosters to provide and sustain tetanus and diphtheria immunity recommended at 4–7 years and 9–15 years [14].) However, outbreaks can still occur in areas with low vaccination rates, and individuals who have not been vaccinated are at risk. For example, according to data from WHO/UNICEF, 14% of the target population had not received the first dose of a DPT-containing vaccine in 2021 (up from 10% in 2019, perhaps related to disruption due to the COVID-19 pandemic) [15, 16]. The prevalence of zero-dose children is particularly high among poorer countries and particularly among families with low socioeconomic status [17]. Only 21% of the target population in low-income countries received the first dose of DPT-containing vaccine in 2021 (17% in 2019) [15]. For example, in Africa, thirty million children suffer from vaccine-preventable diseases annually; of these, more than half a million die [18].

In this paper, we pooled household surveys from 81 low- and middle-income countries conducted in different years between 2014 and 2023 and estimated the percentage of children 12–35 months old who had not received any DPT-containing vaccination. For 68 countries with more than one survey available (with the earlier survey conducted in different years between 2000 and 2013), we also studied the average annual percentage point change in the zero-dose prevalence. Finally, we also explored the association between the levels and trends in zero-dose prevalence, health expenditure, Gavi-eligibility, and postneonatal and child mortality rate. Ensuring full immunization coverage is a foundational function of a healthcare system, and we expect increased spending to correlate with a decline in zero-dose prevalence. Gavi, The Vaccine Alliance, supports the world’s poorest countries in financing vaccinations, and we expect these countries to have a faster decline [19]. Postneonatal and child mortality is a composite child health measure expected to correlate highly with zero-dose prevalence. We excluded neonatal deaths since these are caused primarily by neonatal conditions, such as prematurity, which are unrelated to childhood vaccination status [20].

### Data

We used data from nationally representative cross-sectional household surveys conducted in low- and middle-income countries, specifically the Multiple Indicator Cluster Surveys (MICS) and Demographic and Health Surveys (DHS) [21–24]. The MICS and DHS are highly standardized, and interagency collaboration ensures that the MICS and DHS survey tools are comparable [25]. However, an important difference between the DHS and MICS is that the DHS only collects information on vaccination from biological mothers of children while the MICS also gathers information from caretakers and, consequently, includes foster children. Since the DHS are more numerous and have better standardization of datafiles, we first considered DHS surveys and then only added MICS surveys for countries without a DHS survey. All surveys used were nationally representative, and those restricted to subpopulations or subnational regions were not considered (an exception is “North” Sudan which was surveyed separately before splitting from South Sudan). We included the State of Palestine and Kosovo.

For the first part of our analysis, we considered the most recent survey for each country, limited to countries with a survey conducted after 2014 which resulted in a total of 81 countries: 48 from the DHS and 33 from the MICS (Supplementary Table S1). Our selection of countries was based on the availability of surveys. For the second part of the analysis, we studied the average annual percentage point change for the 68 countries that had at least two surveys: one conducted after 2014 and another before. When more than one survey was available for a country before 2014, we chose the earliest survey. However, we do not consider surveys conducted before 2000. Of the 68 pre-2014 surveys, 50 were from the DHS and 18 from the MICS. All countries were classified as low- or middle-income by the World Bank at the time of the most recent survey [26]. We show survey years in Tables 1 and 2 (end year when surveys were conducted over more than one calendar year) and sample sizes in Supplementary Table S1.

**Table 1:** Zero-dose prevalence and estimated number of zero-dose children

	Survey year	Prevalence (%)	95% confidence interval	Number (thousands)
Pooled	2019*	12.4	12.0, 12.9	21,096
East Asia & Pacific	2019*	11.2	10.4, 12.0	2,306
Cambodia	2022	7.3	6.1, 8.6	46
Fiji	2021	4.3	3.0, 6.1	1.5
Indonesia	2017	10.6	9.5, 11.9	991
Lao	2017	29.4	27.3, 31.6	93
Mongolia	2018	3.8	2.8, 5.2	5.9
Myanmar	2016	13.0	10.4, 16.2	234
Papua New Guinea	2018	36.0	32.7, 39.4	170
Philippines	2022	13.0	11.0, 15.3	627
Samoa	2020	25.8	22.2, 29.8	3.0
Timor-Leste	2016	22.8	20.2, 25.6	14
Tonga	2019	5.1	3.1, 8.3	0.25
Tuvalu	2020	3.8	2.0, 7.0	0.019
Viet Nam	2021	4.3	3.1, 6.0	128
Eastern & Southern Africa	2018*	14.5	13.2, 16.0	4,682
Angola	2016	32.2	29.2, 35.3	692
Burundi	2017	0.9	0.7, 1.3	7.8
Eswatini	2014	4.4	3.2, 5.9	2.7
Ethiopia	2019	29.9	25.0, 35.3	2,051
Kenya	2022	3.5	2.9, 4.2	97
Lesotho	2014	2.1	1.4, 3.1	2.3
Madagascar	2021	22.6	20.2, 25.1	378
Malawi	2016	2.8	2.2, 3.4	32
Mozambique	2015	11.7	9.1, 15.0	221
Rwanda	2020	0.5	0.3, 0.8	3.6
South Africa	2016	10.5	8.4, 13.2	245
Sudan	2014	17.7	15.4, 20.4	430
Tanzania	2022	5.2	4.3, 6.3	227
Uganda	2016	5.9	5.1, 6.8	165
Zambia	2019	2.3	1.7, 3.1	27
Zimbabwe	2015	12.0	10.0, 14.4	111
Europe & Central Asia	2018*	7.2	6.1, 8.6	361
Armenia	2016	2.6	1.6, 4.4	2.3
Kazakhstan	2015	3.5	2.7, 4.6	27
Kosovo	2020	3.9	2.6, 5.9	1.5
Kyrgyzstan	2018	7.1	5.5, 9.2	23
North Macedonia	2019	3.2	1.6, 6.2	1.4
Serbia	2019	4.4	2.7, 7.0	6.1
Tajikistan	2017	7.4	6.0, 9.2	38
Turkmenistan	2016	0.4	0.2, 0.9	1.2
Türkiye	2019	9.4	7.4, 11.8	262
Latin America & Caribbean	2016*	6.8	5.5, 8.4	505
Belize	2016	7.2	5.0, 10.4	1.1
Costa Rica	2018	2.6	1.6, 4.0	3.6
Cuba	2019	3.2	2.0, 4.9	7.3
Dominican Republic	2019	10.0	8.7, 11.5	41
El Salvador	2014	1.1	0.7, 1.8	2.6
Guatemala	2015	2.0	1.6, 2.6	16
Guyana	2014	3.6	2.5, 5.2	1.1
Haiti	2017	18.4	15.8, 21.3	93
Honduras	2019	3.4	2.6, 4.3	14
Mexico	2015	7.1	5.1, 10.0	312
Paraguay	2016	4.3	3.4, 5.6	12
Suriname	2018	22.8	19.5, 26.3	4.9
Middle East & North Africa	2016*	4.9	4.5, 5.4	515
Algeria	2019	5.2	4.4, 6.1	103
Egypt	2014	0.8	0.5, 1.2	40
Iraq	2018	13.1	11.6, 14.7	298
Jordan	2018	7.8	6.3, 9.7	37
State of Palestine	2020	4.6	3.6, 5.9	13
Tunisia	2018	4.6	3.5, 6.0	20
South Asia	2020*	8.6	8.0, 9.2	5,718
Afghanistan	2015	31.7	27.9, 35.8	742
Bangladesh	2018	1.5	1.0, 2.3	87
India	2021	6.7	6.4, 7.0	3,056
Maldives	2017	9.3	7.0, 12.3	1.4
Nepal	2022	6.1	4.8, 7.6	72
Pakistan	2018	15.3	12.5, 18.7	1,789
West & Central Africa	2019*	26.0	24.8, 27.3	7,178
Benin	2018	16.5	14.6, 18.6	134
Burkina Faso	2021	5.9	4.8, 7.3	85
Cameroon	2019	16.9	14.5, 19.5	288
Chad	2015	43.1	40.1, 46.1	488
Congo	2015	14.7	12.8, 16.8	49
Cote d’Ivoire	2021	30.0	27.5, 32.7	507
Gabon	2021	16.1	13.7, 18.8	20
Gambia	2020	2.1	1.5, 2.9	3.5
Ghana	2023	3.5	2.7, 4.5	62
Guinea	2018	36.8	33.3, 40.4	301
Guinea-Bissau	2019	8.5	7.1, 10.3	10.0
Liberia	2020	9.7	7.7, 12.1	29
Mali	2018	18.1	15.6, 20.8	273
Mauritania	2021	12.8	11.2, 14.5	36
Nigeria	2018	36.3	34.1, 38.5	4,864
Sao Tome and Principe	2019	4.3	3.0, 6.1	0.52
Senegal	2019	4.1	2.9, 5.7	41
Sierra Leone	2019	5.8	4.8, 7.0	27
Togo	2017	10.3	8.5, 12.5	50

*Note* *Average survey year is shown for pooled and regional estimates. The number of zero-dose children was estimated using the zero-dose prevalence and an estimate of the population of children 12–35 months old obtained from the United Nation World Population Prospects linked to the country and year of survey. Estimates refer to the latest survey in each country. 95% confidence intervals (CI) were adjusted for clustering at the level of primary sampling units. Estimates were weighted using sampling weights rescaled to sum up the population 12–35 months old in the country and year of survey

**Table 2:** Zero-dose prevalence and average annual percentage point (pp) change in prevalence

	Earlier Survey			Later Survey			Average annual change	
	Survey year	Zero-dose	95% CI	Survey year	Zero-dose	95% CI	Change	95% CI
		%	%		%	%	pp	%
Pooled	2005**	22.5	21.7, 23.3	2019**	12.3	11.9, 12.8	-0.7*	-0.8, -0.6
East Asia & Pacific	2002**	15.8	14.4, 17.2	2019**	10.6	9.8, 11.5	-0.3*	-0.4, -0.2
Cambodia	2000	34.2	30.9, 37.5	2022	7.3	6.1, 8.6	-1.2*	-1.4, -1.1
Indonesia	2003	19.5	17.0, 22.3	2017	10.6	9.5, 11.9	-0.6*	-0.8, -0.4
Lao	2000	36.6	32.6, 40.7	2017	29.4	27.3, 31.6	-0.4*	-0.7, -0.2
Mongolia	2000	6.4	4.8, 8.5	2018	3.8	2.8, 5.2	-0.1*	-0.3, -0.0
Myanmar	2000	10.1	8.8, 11.6	2016	13.0	10.4, 16.2	0.2	-0.0, 0.4
Philippines	2003	10.5	9.1, 12.1	2022	13.0	11.0, 15.3	0.1	-0.0, 0.3
Timor-Leste	2010	25.6	23.1, 28.4	2016	22.8	20.2, 25.6	-0.5	-1.1, 0.2
Viet Nam	2002	9.9	6.8, 14.2	2021	4.3	3.1, 6.0	-0.3*	-0.5, -0.1
Eastern & Southern Africa	2003**	24.9	23.5, 26.4	2018**	14.8	13.4, 16.4	-0.7*	-0.8, -0.5
Angola	2001	45.7	42.6, 48.8	2016	32.2	29.2, 35.3	-0.9*	-1.2, -0.6
Burundi	2011	1.1	0.7, 1.5	2017	0.9	0.7, 1.3	-0.0	-0.1, 0.1
Eswatini	2007	3.3	2.3, 4.8	2014	4.4	3.2, 5.9	0.1	-0.1, 0.4
Ethiopia	2000	53.8	50.3, 57.4	2019	29.9	25.0, 35.3	-1.3*	-1.6, -0.9
Kenya	2009	5.3	4.0, 7.0	2022	3.5	2.9, 4.2	-0.1*	-0.3, -0.0
Lesotho	2005	6.1	4.6, 7.9	2014	2.1	1.4, 3.1	-0.4*	-0.6, -0.2
Madagascar	2004	27.5	21.9, 33.9	2021	22.6	20.2, 25.1	-0.3	-0.7, 0.1
Malawi	2000	3.7	3.0, 4.6	2016	2.8	2.2, 3.4	-0.1	-0.1, 0.0
Mozambique	2004	14.0	11.8, 16.7	2015	11.7	9.1, 15.0	-0.2	-0.6, 0.1
Rwanda	2000	5.2	4.3, 6.3	2020	0.5	0.3, 0.8	-0.2*	-0.3, -0.2
Sudan	2000	32.2	30.2, 34.3	2014	17.7	15.4, 20.4	-1.0*	-1.3, -0.8
Tanzania	2005	6.8	5.2, 9.0	2022	5.2	4.3, 6.3	-0.1	-0.2, 0.0
Uganda	2001	20.5	17.8, 23.4	2016	5.9	5.1, 6.8	-1.0*	-1.2, -0.8
Zambia	2002	6.0	4.9, 7.5	2019	2.3	1.7, 3.1	-0.2*	-0.3, -0.1
Zimbabwe	2006	26.9	23.8, 30.2	2015	12.0	10.0, 14.4	-1.7*	-2.1, -1.2
Europe & Central Asia	2007**	7.8	6.3, 9.5	2018**	7.7	6.5, 9.2	-0.0	-0.2, 0.2
Armenia	2000	7.1	5.0, 9.9	2016	2.6	1.6, 4.4	-0.3*	-0.5, -0.1
Kazakhstan	2011	1.5	0.9, 2.4	2015	3.5	2.7, 4.6	0.5*	0.2, 0.8
Kosovo	2014	2.6	1.5, 4.5	2020	3.9	2.6, 5.9	0.2	-0.1, 0.6
Kyrgyzstan	2012	1.8	1.1, 2.9	2018	7.1	5.5, 9.2	0.9*	0.5, 1.2
Serbia	2006	4.3	3.2, 5.7	2019	4.4	2.7, 7.0	0.0	-0.2, 0.2
Tajikistan	2012	3.8	2.8, 5.0	2017	7.4	6.0, 9.2	0.7*	0.3, 1.1
Türkiye	2004	11.3	9.0, 14.2	2019	9.4	7.4, 11.8	-0.1	-0.4, 0.1
Latin America & Caribbean	2004**	7.2	6.0, 8.6	2018**	9.2	8.3, 10.2	0.1*	0.0, 0.2
Belize	2011	6.9	5.1, 9.3	2016	7.2	5.0, 10.4	0.1	-0.6, 0.8
Costa Rica	2011	1.9	0.9, 3.7	2018	2.6	1.6, 4.0	0.1	-0.2, 0.3
Cuba	2006	0.5	0.2, 0.9	2019	3.2	2.0, 4.9	0.2*	0.1, 0.3
Dominican Republic	2002	5.6	4.7, 6.6	2019	10.0	8.7, 11.5	0.3*	0.2, 0.4
Guyana	2009	8.8	6.7, 11.3	2014	3.6	2.5, 5.2	-1.0*	-1.6, -0.5
Haiti	2000	20.6	16.7, 25.1	2017	18.4	15.8, 21.3	-0.1	-0.4, 0.2
Honduras	2006	1.0	0.6, 1.4	2019	3.4	2.6, 4.3	0.2*	0.1, 0.3
Suriname	2001	10.1	7.7, 13.3	2018	22.8	19.5, 26.3	0.7*	0.5, 1.0
Middle East & North Africa	2006**	4.7	4.3, 5.1	2016**	4.9	4.5, 5.4	0.0	-0.0, 0.1
Algeria	2013	3.3	2.7, 4.0	2019	5.2	4.4, 6.1	0.3*	0.1, 0.5
Egypt	2000	0.8	0.5, 1.2	2014	0.8	0.5, 1.2	-0.0	-0.0, 0.0
Iraq	2011	14.1	13.0, 15.2	2018	13.1	11.6, 14.7	-0.1	-0.4, 0.1
Jordan	2002	0.4	0.2, 0.8	2018	7.8	6.3, 9.7	0.5*	0.4, 0.6
State of Palestine	2010	3.2	2.6, 3.9	2020	4.6	3.6, 5.9	0.1*	0.0, 0.3
Tunisia	2013	1.4	0.9, 2.4	2018	4.6	3.5, 6.0	0.6*	0.3, 0.9
South Asia	2006**	23.3	22.1, 24.5	2020**	7.7	7.1, 8.4	-1.1*	-1.2, -1.0
Bangladesh	2004	6.9	5.4, 8.9	2018	1.5	1.0, 2.3	-0.4*	-0.5, -0.3
India	2006	25.1	23.7, 26.6	2021	6.7	6.4, 7.0	-1.2*	-1.3, -1.1
Maldives	2009	0.8	0.4, 1.5	2017	9.3	7.0, 12.3	1.1*	0.7, 1.4
Nepal	2001	15.2	12.1, 18.9	2022	6.1	4.8, 7.6	-0.4*	-0.6, -0.3
Pakistan	2007	25.8	23.5, 28.3	2018	15.3	12.5, 18.7	-1.0*	-1.3, -0.6
West & Central Africa	2004**	37.2	34.3, 40.2	2019**	26.0	24.8, 27.3	-0.8*	-1.0, -0.6
Benin	2001	13.1	10.6, 15.9	2018	16.5	14.6, 18.6	0.2*	0.0, 0.4
Burkina Faso	2003	23.2	19.9, 26.9	2021	5.9	4.8, 7.3	-1.0*	-1.2, -0.8
Cameroon	2004	17.1	14.8, 19.7	2019	16.9	14.5, 19.5	-0.0	-0.2, 0.2
Chad	2004	56.6	50.7, 62.2	2015	43.1	40.1, 46.1	-1.2*	-1.8, -0.6
Congo	2005	14.2	11.4, 17.6	2015	14.7	12.8, 16.8	0.0	-0.3, 0.4
Cote d’Ivoire	2012	20.6	17.8, 23.8	2021	30.0	27.5, 32.7	1.0*	0.6, 1.5
Gabon	2001	26.5	23.6, 29.5	2021	16.1	13.7, 18.8	-0.5*	-0.7, -0.3
Gambia	2013	2.6	1.9, 3.6	2020	2.1	1.5, 2.9	-0.1	-0.2, 0.1
Ghana	2003	9.9	8.0, 12.3	2023	3.5	2.7, 4.5	-0.3*	-0.4, -0.2
Guinea	2005	22.9	20.0, 26.2	2018	36.8	33.3, 40.4	1.1*	0.7, 1.4
Guinea-Bissau	2000	26.5	26.5, 26.5	2019	8.5	7.1, 10.3	-0.9*	-1.0, -0.9
Liberia	2007	25.2	20.2, 30.8	2020	9.7	7.7, 12.1	-1.2*	-1.6, -0.7
Mali	2001	37.3	34.3, 40.5	2018	18.1	15.6, 20.8	-1.1*	-1.4, -0.9
Mauritania	2011	13.1	11.5, 15.0	2021	12.8	11.2, 14.5	-0.0	-0.3, 0.2
Nigeria	2003	56.0	50.8, 61.0	2018	36.3	34.1, 38.5	-1.3*	-1.7, -0.9
Sao Tome and Principe	2009	6.8	4.9, 9.4	2019	4.3	3.0, 6.1	-0.3	-0.5, 0.0
Senegal	2005	7.8	6.7, 9.2	2019	4.1	2.9, 5.7	-0.3*	-0.4, -0.1
Sierra Leone	2008	24.0	21.2, 27.1	2019	5.8	4.8, 7.0	-1.7*	-1.9, -1.4
Togo	2014	7.3	5.8, 9.2	2017	10.3	8.5, 12.5	1.0*	0.1, 1.9

*Note* *p < 0.05. **Average survey year is shown for pooled and regional estimates. 95% confidence intervals (CI) were adjusted for clustering at the level of primary sampling units. Estimates were weighted using sampling weights rescaled to sum up the population 12–35 months old in the country and year of survey

The DHS and MICS surveys generally use stratified multi-stage sampling, which involves sampling primary sampling units (such as villages or neighborhoods) from strata of geographic or administrative subnational regions, further split into urban and rural areas, with a probability proportional to size. Then around 20–30 households are sampled from each primary sampling unit using systematic random sampling. Women aged 15–49 are interviewed in these households, with questions about their birth histories, health, and children’s health. Response rates tend to exceed 90% in these surveys [21, 23]. Sampling weights are calculated to adjust for non-responses and oversampling and to improve precision [23, 27]. In most surveys, mothers (or caretakers) provide information on their children’s vaccinations. Vaccine information is typically gathered for children under three or five years old; we restrict all surveys to children under 36 months old. In one MICS survey, the upper age limit was 24 months: Cuba in 2006. We also excluded children less than 12 months old to allow some delay in receiving the first dose of DPT (e.g., in relation to vaccination campaigns).

When only considering the most recent survey for each country, our total sample consisted of 345 660 children aged 12–35 months (Supplementary Table S1). For the 68 countries with more than one survey, and therefore used to estimate the average annual change, the final sample in the earlier surveys consisted of 207 687 children 12–35 months.

We retrieved yearly data on neonatal and under-five mortality rates (deaths per 1000 live births), prepared by the United Nations Inter-agency Group for Child Mortality Estimation, from the World Bank Development Indicators [28]. We calculated postneonatal and child mortality rate (i.e., mortality from age 28 days to 59 months) by subtracting the neonatal mortality rate from the under-five mortality rate.

We used data on health expenditure from the World Health Organization Global Health Expenditure database [29], retrieved from the World Bank Development Indicators [28]. We retrieved annual series on current health expenditure (Current health expenditure [% of GDP]), which shows all current spending on health (i.e., including healthcare goods and services but excluding capital health expenditures) within the country as a share of GDP. We retrieved GDP per capita (GDP per capita, PPP [constant 2017 international $]) from the World Bank Development indicators [28]. We used these two measures—GDP and current health expenditure as a percentage of GDP—to calculate current health expenditure per capita in PPP adjusted constant 2017 international $.

We also obtained age-specific population data from the 2022 United Nations World Population Prospects [30], which we used to construct a variable indicating the relative size of the under-five population, or the percentage of the total population under age five. GDP and relative size of the under-five population were only used as control variables in regression models. Gaps in these time series of three years or less were interpolated or extrapolated linearly. Gaps in these time series of more than three years were not considered for analysis involving these variables (Cuba had missing GDP, the State of Palestine and Kosovo had missing health expenditure data, and Zimbabwe had missing health expenditure data for the earlier survey).

## Methods

We first defined a binary variable for zero-dose, indicating whether a child 12–35 months old received any DPT-containing vaccine (e.g., DPT, Pentavalent, DTC, Hexavalent). The survey year was defined by the end year when a survey spanned more than one calendar year.

For each survey, the percentage of children who did not receive any DPT-containing vaccine (the prevalence of zero-dose children) was calculated. In a few cases, children who were indicated not to have received (or had missing information) on the first dose had valid information indicating that they had received the second or third dose: These children were included in the analysis and not considered zero-dose. We coded children with missing vaccine information or a mother who reported that she did not know whether the child received a DPT vaccine as zero-dose (we show results excluding these as sensitivity analyses, discussed below).

We tabulated the prevalence of zero-dose children for each country in the most recent survey. We also tabulated the prevalence by region and for the pooled sample. We estimated how many children did not receive any DPT-containing vaccine (referred to as number of zero-dose children) in each country using our estimated percentage of zero-dose children and the United Nations World Population Prospects [30] estimates of the population of children 12–35 months in each country and year of survey. Population data was only available until 2021, so 2022 and 2023 were linearly extrapolated for countries surveyed in these years.

### Average Annual Change in zero-dose Prevalence

Further, for 68 countries with more than one survey, we estimated the average annual percentage point change in zero-dose prevalence using linear regression models, by regressing the binary outcome for zero-dose prevalence on the year of survey (end year when surveys spanned more than one calendar year), including two surveys from each country. When estimating the average annual percentage point change for regions and the pooled sample, we regressed the zero-dose indicator on the (weighted) average survey year. Using this approach, the coefficients for the survey year are identical to subtracting the prevalence in the later survey from the prevalence in the earlier survey and dividing by the number of years between surveys. We then show the relationship between the zero-dose prevalence in the earliest survey and the average annual percentage point change to assess the extent of convergence between countries.

The construction of all estimates from the survey data was weighted using sampling weights that were rescaled to sum up the population of children 12–35 months in each country and year of survey, using population estimates for children 12–35 months from the United Nations World Population Prospects (2022 and 2023 were linearly extrapolated when needed) [30]. All 95% confidence intervals were based on standard errors adjusted for clustering at the level of primary sampling units. Confidence intervals for prevalence measures were adjusted using logit transformation to ensure an interval above 0 and below 100.

### Correlates of Levels and Trends in Zero-Dose Prevalence

The average annual changes in GDP, health expenditure, postneonatal and child mortality rates, and the relative size of the under-five population were done in an equivalent way as for zero-dose prevalence. GDP and health expenditure were converted to natural log scale. Only one country changed Gavi-eligibility between the earlier and later survey: therefore, we used eligibility in the earlier survey when analyzing average annual change, rather than change across surveys (Supplementary Table S1).

We plot the zero-dose prevalence against the variables for health expenditure and postneonatal and child mortality rate, both the levels in the most recent survey and the average annual change, and show the Pearson’s correlation coefficients.

Finally, we explored whether there was an association between health expenditure, Gavi eligibility, and zero-dose prevalence, as well as zero-dose prevalence and postneonatal and child mortality rate, independent of GDP per capita and relative size of the under-five population (since a large child-population can strain resources for vaccination efforts). Note, however, that our approach does not determine causality. To do this, we run two linear regression models: First, we regress zero-dose prevalence on health expenditure and a binary indicator for Gavi-eligibility, while controlling for GDP, the relative size of the under-five population, and the survey year. The regressions show the association of health expenditure and Gavi-eligibility with zero-dose prevalence, independent of each other, as well as of GDP, survey year, and the relative size of the under-five population.

Second, we regress postneonatal and child mortality rate on zero-dose prevalence, health expenditure, GDP, relative size of the under-five population, and survey year. Similarly, this regression shows the association between zero-dose prevalence and mortality independent of GDP, health expenditure, the relative size of the under-five population, and year of survey. We do these regressions both for the most recent survey only and another for the average annual change in all measures (except for the year of survey and Gavi-eligibility which indicates Gavi eligibility in the earlier survey). Since Gavi-eligibility is determined by level of aggregate income, we do not include Gavi-eligibility in the regression using levels (which also includes the level of GDP), only those using average annual change. Using average annual change instead of levels has the advantage of further adjusting for all country-level factors that were constant between surveys. Robust standard errors are shown and used to calculate p-values.

### Supplementary and Sensitivity Analyses

As a supplementary analysis, we also show distribution of responses to the DPT vaccination questions: (1) missing DPT vaccination information, (2) children whose mothers reported that they did not know whether their child received a DPT vaccine, (3) children whose mothers reported that the child was not vaccinated with DPT, 3) children whose mothers reported from recall that their child was vaccinated with DPT, (4) children whose mothers reported that their child was vaccinated with DPT based on information on a vaccination card (Supplementary Table S3). We also report Pearson’s correlation of zero-dose prevalence with the share of vaccinations reported by the mother (as opposed to from a card) and the share of children with unknown (by mother) or missing vaccine information.

We also redo our analyses while excluding parts of our sample. First, we excluded (i.e., code as missing), children whose information on DPT vaccinations was missing: that is, the question was unanswered or not coded in the dataset (Supplementary Tables S4–7 and Figures S1–3). Second, in addition to missing information, we also excluded children whose mothers answered that they did not know whether their child had received a DPT vaccine (Supplementary Tables S8–11 and Figures S4–6). Since we only remove children who were defined as zero-dose before in these two sensitivities analyses, the prevalence of zero-dose will inevitably be reduced. We discuss how these exclusions impact the average annual change.

## Results

**Fig. 1:**
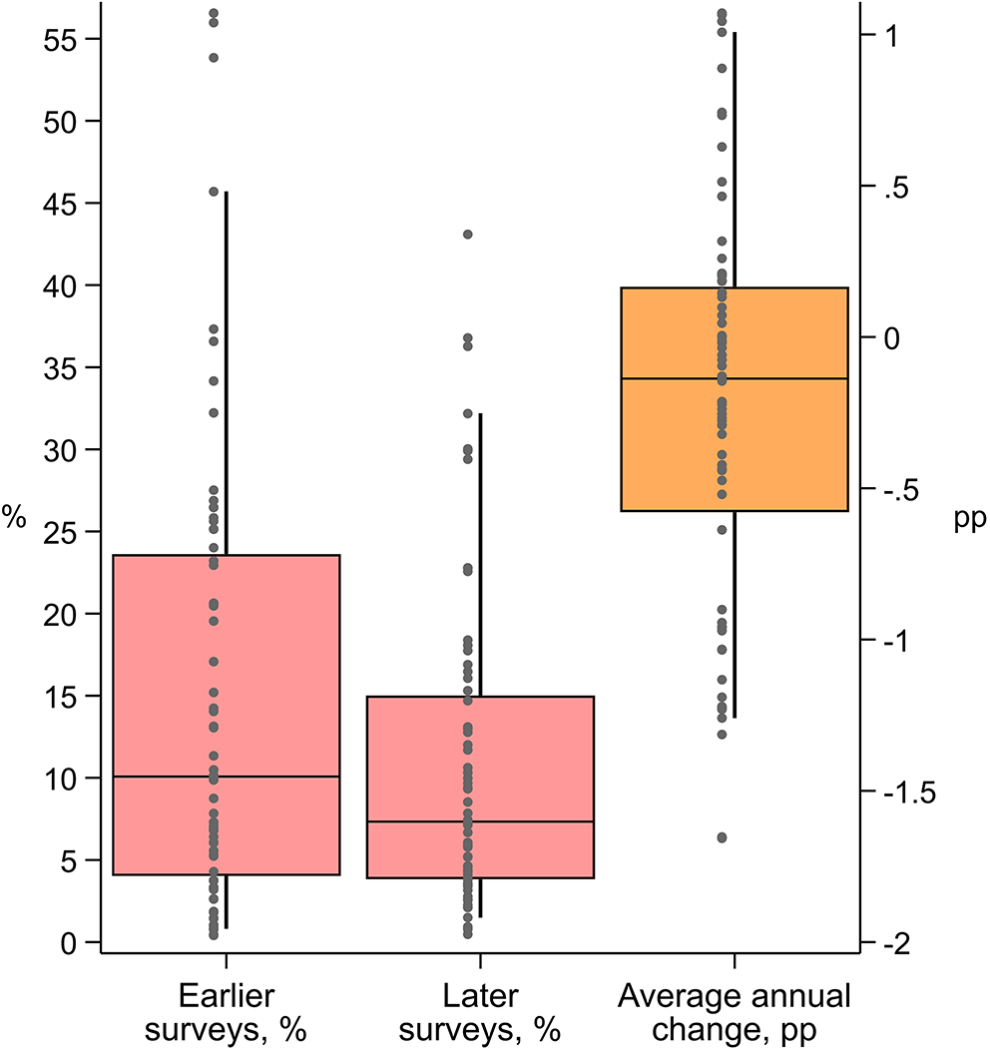
Distribution of zero-dose prevalence across countries by survey year. *Note* Only includes countries with two surveys. Percentiles 5 and 95 (line) and 25, 50, and 75 (box) are shown. Dots indicate country estimates. Each country’s estimate was weighted using sampling weights. Surveys were equally weighted for the median and percentiles. See Table 2 and S2 for tabulated estimates. Percentage point (pp) average annual change (AAC) is shown on the right side y-axis

For countries with two surveys, the median indicated that 10% (interquartile range [IQR] 4, 24) of children 12–35 months had not received a dose of DPT-containing vaccine, which decreased to 7% (IQR 4, 15) in the later surveys (Fig. 1). The median country’s average annual change (AAC) was − 0.14% points (IQR − 0.6, 0.17). Note that countries were equally weighted.

In the pooled sample, where sampling weights were rescaled to reflect population size, 12% of children had not received a DPT-containing vaccine, or an estimated just over 21 million children across all 81 countries (Table 1). The prevalence of zero-dose children was the highest in West & Central Africa, 26%, or an estimated 7 million children across countries included in our sample. Eastern & Southern Africa had the second highest zero-dose prevalence, 15%, or almost 5 million children across countries included in our sample. The second lowest prevalence was observed in Latin America & Caribbean region, 7%, or 500 000 children across all countries in our sample. The lowest prevalence was observed in Middle East & North Africa 5% (estimated around 500 000 children). South Asia had a prevalence of 9% (estimated around 6 million children), Europe & Central Asia, 7%, (or around 400 000 children), and East Asia & Pacific 11% (estimated over 2 million children).

The highest prevalence was observed in Chad (2015), 43% (estimated almost 500 000 children) and Nigeria (2018), 36% (estimated close to 5 million children). The lowest zero-dose prevalence was observed in Turkmenistan (2016), 0.4% (estimated around 1200 children) and El Salvador (2014), 1.1 (estimated 2 600 children).

### Average Annual Percentage Point Change

In the pooled sample of 68 countries with two surveys, the prevalence of zero-dose children was 23% in the earlier surveys (with an average survey year of 2005), which declined to 12% in the later surveys (conducted in 2019 on average), or a 0.7 average annual percentage point decline over the period (Table 2). Of the seven global regions, South Asia had the fastest decline in zero-dose prevalence, or 1.1% point average annual decline.

The largest average annual decline was observed in Zimbabwe (2006 to 2015), at 1.7% points; and Sierra Leone (2008 to 2019), at 1.7% points. A statistically significant increase in the prevalence of zero-dose children was observed in several countries. The largest increase was observed in the Maldives (2009 to 2017), 1.1% point increase per year on average, Guinea (2005 to 2018), 1.1% points, and Cote d’Ivoire (2012 to 2021), 1% points.

**Fig. 2:**
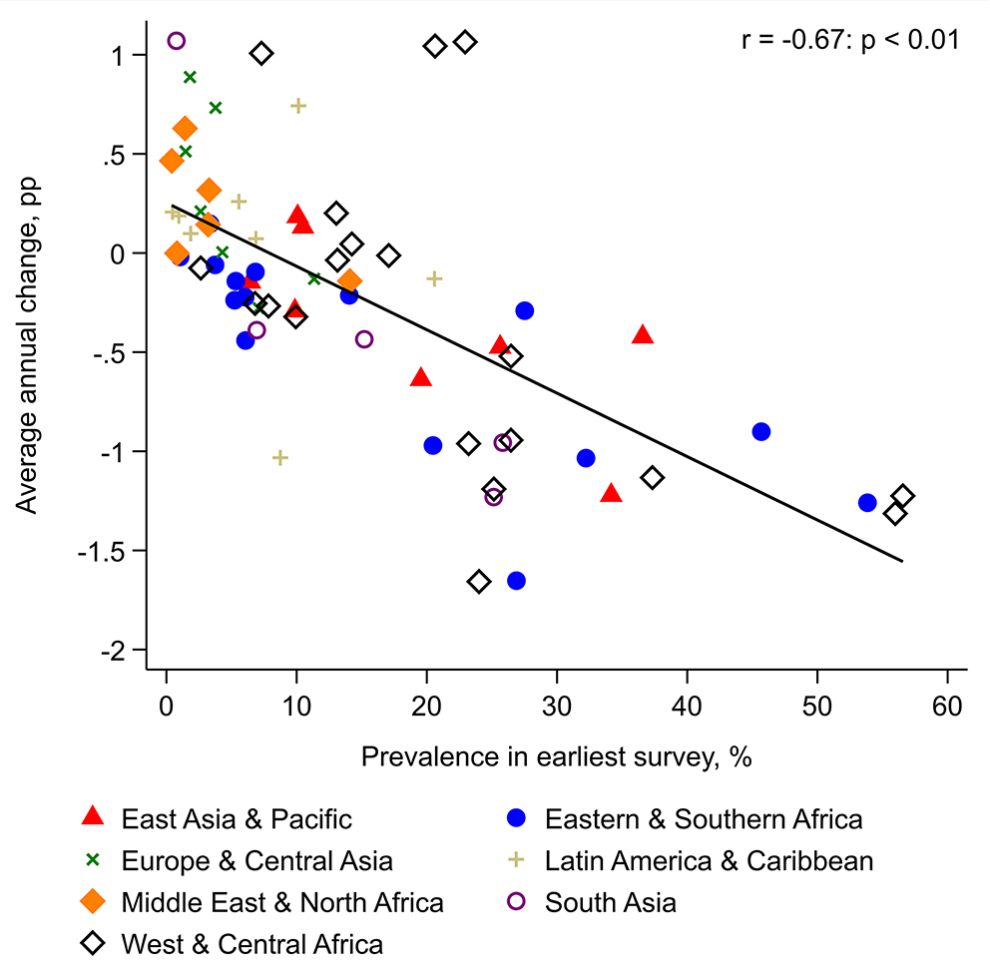
The relationship between country-level zero-dose prevalence in the earliest survey and average annual percentage point (pp) change in prevalence. Pearson’s correlation coefficient (r) is shown. Countries were equally weighted.

Overall, there was a strong negative correlation (-0.67: *p* < 0.01) between the prevalence of zero-dose children in the earliest survey and the average annual percentage point change between the earliest and latest surveys, indicating convergence across countries (Fig. 2).

### Relationship between zero-dose Prevalence and Macro-Level Variables

**Fig. 3:**
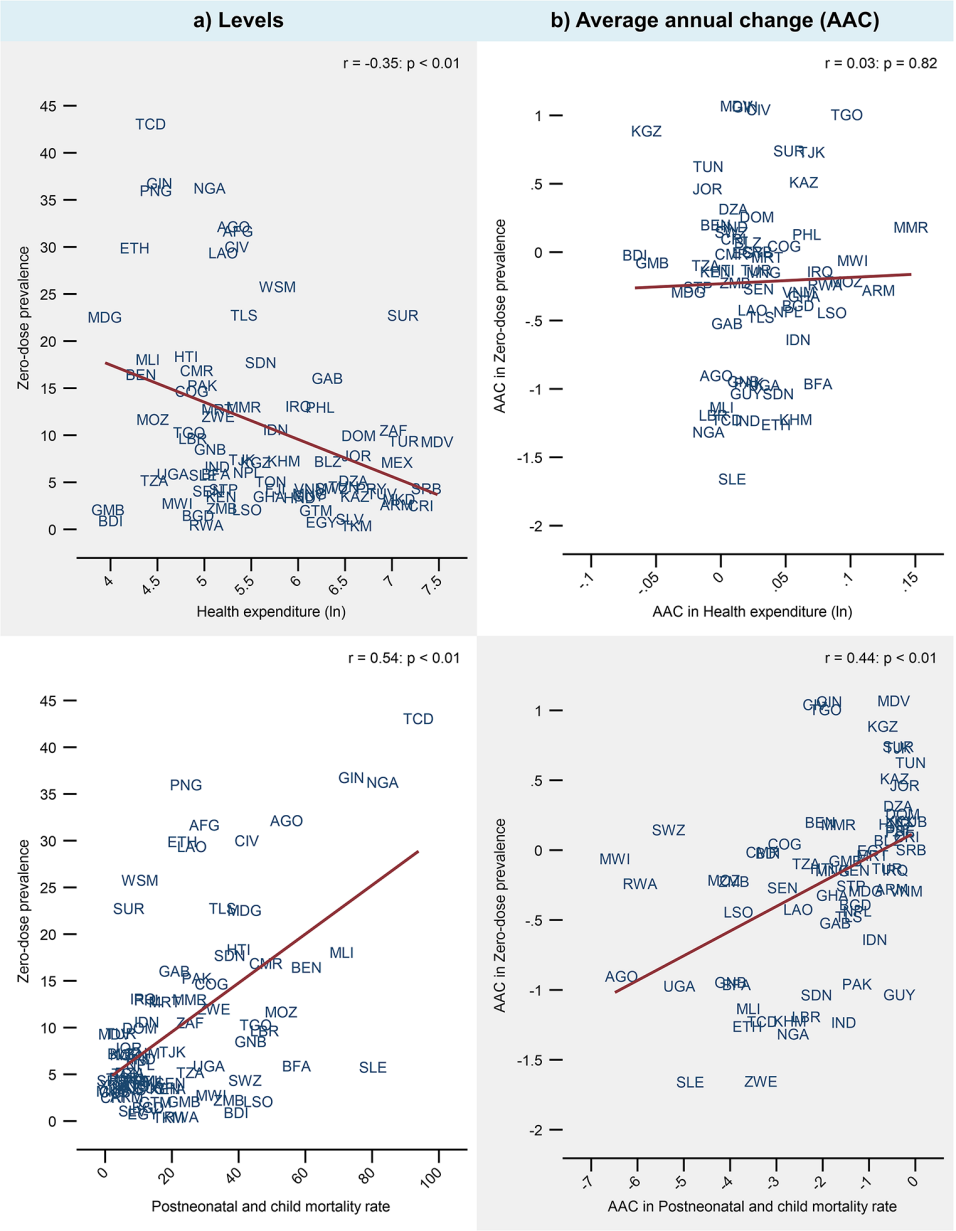
Correlation of zero-dose prevalence with health expenditure and postneonatal and child mortality rate. *Notes* The y and x-axes vary across graphs. GDP and health expenditure were measured per capita in PPP adjusted constant 2017 international $. Pearson’s correlation coefficients (r) are shown. Postneonatal and child mortality rate is deaths per 1,000 live births. Countries were equally weighted

There was a moderate negative correlation between the level of zero-dose prevalence and health expenditure per capita (*r*=-0.35: *p* < 0.01: Fig. 3). On the other hand, there was no correlation between the average annual change in health expenditure and the average annual change in zero-dose prevalence (*r* = 0.03: *p* = 0.8).

After adjusting for GDP per capita, relative size of the under-five population, and year of survey, there was a strong positive association between level of health expenditure and zero-dose prevalence. A single log point increase in health expenditure was associated with a 8% points lower zero-dose prevalence (or around 0.8% points for a 10% increase in expenditure: Table 3). No statistically significant association was found between the average annual change in zero-dose prevalence and health expenditure. Gavi-eligible countries had a 0.57 faster average annual percentage point change in zero-dose prevalence than non-Gavi-eligible countries (*p* < 0.01).

**Table 3:** Linear regressions

	(1)	(2)	(3)	(4)
	Level in latest survey		Average annual change	
	Zero-dose prevalence	Postneonatal and child mortality	Zero-dose prevalence	Postneonatal and child mortality
Zero-dose prevalence		0.59*		1.23*
		(0.24)		(0.27)
Total health expenditure (ln)	-8.10*	-1.62	2.02	-7.42
	(2.82)	(4.53)	(2.85)	(7.68)
GDP (ln)	7.57*	-7.10	-0.34	9.92
	(2.77)	(4.71)	(6)	(12.27)
Population below age 5 (%)	-0.24*	-0.50*	0.06	-0.15
	(0.09)	(0.13)	(0.03)	(0.09)
Survey year	-0.04	-0.77	-0	0.12
	(0.41)	(0.52)	(0.03)	(0.09)
Gavi-eligible			-0.57*	
			(0.14)	
Constant	11.05*	25.52*	-0.22*	-2.03*
	(0.99)	(1.42)	(0.08)	(0.19)
R squared	0.30	0.67	0.19	0.26
Observations	78	78	64	64

Note *P < 0.05. Dependent variables are indicated at the top of each column. In columns 3 and 4, all variables are expressed as average annual absolute change, except for survey year and Gavi-eligibility. All values were mean-centered. GDP and health expenditure are per capita in PPP adjusted constant 2017 international $. Postneonatal and child mortality rate is expressed as deaths per 1,000 live births. Countries were equally weighted. Robust standard errors are shown in parentheses below coefficients

### Relationship Between Zero-Dose Prevalence and Postneonatal and Child Mortality Rate

There was a positive correlation between the level of zero-dose prevalence and postneonatal and child mortality rate (*r* = 0.54: *p* < 0.01: Fig. 3). There was further a positive correlation between the average annual change in zero-dose prevalence and change in postneonatal and child mortality rate (*r* = 0.4: *p* < 0.01). After adjusting for GDP, health expenditure, relative size of the under-five population, and survey year, a single percentage point greater level of zero-dose prevalence was associated with a 0.6 greater postneonatal and child deaths per 1000 births (Table 3). Making the same adjustments, a single percentage point greater average annual increase in zero-dose prevalence was associated with an average annual increase of 1.2 deaths per 1000 births.

### Supplementary and Sensitivity Analyses

In the pooled sample, 88% of children had DPT vaccination—23% reported by the mother from memory and 64% from a vaccination card—11% had no vaccination reported, 0.7% had mothers who responded that they did not know whether the child received DPT, and 0.5% had missing vaccine information (Supplementary Table S3). As many as 62% of children had vaccinations reported by their mothers from memory and as many as 16% had either missing information on vaccinations or mothers who did not recall whether the child received a DPT vaccine. In general, a higher share of immunization was reported by mothers (for children who received a vaccine) in countries with a higher prevalence of zero-dose children (*r* = 0.5: *p* < 0.01). A small positive correlation was found between zero-dose prevalence and the share of children with missing vaccine information or mothers who answered that they did not recall whether the child received a vaccine (*r* = 0.2: *p* < 0.1).

When excluding children with missing vaccine information (rather than coding them as zero-dose) there was generally a lower zero-dose prevalence (Supplementary Table S5). However, the average annual change was similar overall, although there were considerable changes in some contexts, which tended show attenuated change across time (Supplementary Figure S1 and Table S6). Further, the link between average annual change in postneonatal and child mortality and zero-dose prevalence attenuated somewhat after excluding children with missing vaccine information (Figure S3 and Table S7). Overall, the results were similar when further excluding children whose mothers reported that they did not know whether the child received a DPT vaccine (Supplementary Tables S8–11 and Figures S4–6).

## Discussion

Not receiving the first dose of a DPT-containing vaccine is widely used as a proxy for “zero-dose” children—ie, children who do not receive any routine vaccinations. This paper estimated the percentage of children not receiving a dose of a DPT-containing vaccine in 81 low- and middle-income countries. We estimated a prevalence of zero-dose children of 12% when pooling the most recent surveys across countries. We find a substantial decline overall, particularly among countries with high prevalence and Gavi-eligible countries. We further found a robust association between zero-dose prevalence and postneonatal and child mortality rate. The link between health expenditure per capita and zero-dose prevalence was less robust.

Studies have suggested a high return from investing in vaccinations in terms of averting treatment costs and productivity losses, or more than 16–44 times the cost (considering 10 common antigens) [31]. National mass vaccination campaigns have successfully been implemented and could help reduce zero-dose prevalence [32]. However, the integration of immunization into other healthcare delivery is important. Receiving the first vaccine is highly correlated with receiving all vaccines [5]. Zero-dose children and their mothers have also been found to be substantially less likely to receive other primary healthcare interventions, such as the recommended number of antenatal care visits and institutional delivery [8]. Studies have also found that zero-dose children tend to have worse living standards in terms of access to water, sanitation, education, and general deprivation [7], exposing them to multiple risk simultaneously.

The level of current health expenditure per capita was only moderately correlated with zero-dose prevalence, and no correlation was found for changes across time, suggesting that health expenditure may be neglecting vaccinations, despite it being a foundational health intervention. It should be noted that bias in immunization data from non-random missingness or recall bias may attenuate the true correlation with health expenditure. However, other studies have also found mixed results on the links between health expenditure and other vaccine indicators. The critical role of government expenditure on health combined with political will has been credited with successes in expanding vaccination coverage, for example, in Rwanda [33, 34]. UNICEF has noted an alarming decline in confidence in childhood vaccines in many countries, which might to an extent explain the weak link between health spending and zero-dose prevalence [35].

The main difference between the estimates in our paper and estimates of vaccine status reported in the Global Burden of Disease (GBD) study [36] is that ours are directly estimated from household surveys. GBD estimates were modeled using various data sources, such as household surveys and country-reported coverage data. Direct survey estimates have greater transparency and reproducibility. Household surveys like the DHS and MICS collect data through well-defined methodologies and provide data with well-known limitations. These surveys have well-established protocols, questionnaires, and sampling strategies, ensuring transparency in data collection and quality control measures, accompanied by detailed documentation. These surveys have been conducted over many years, covering numerous countries, and have become widely recognized as essential sources of health-related data, including vaccine coverage, in low- and middle-income countries. Since these surveys are highly standardized, researchers can easily extend this study when new surveys become available.

### Limitations

This study had several limitations. First, data coverage of low- and middle-income countries was incomplete. Some regions were covered more extensively than others, which should be considered when interpreting these results. Coverage of upper-middle-income countries was especially lacking (for example, no data was available for China, Brazil, and Russia). However, our sample included almost 70% of children aged 1–2 years old in low- and middle-income countries [30].

Second, the vaccination status was reported by mothers from memory when the mother did not possess a vaccination card, which may suffer from inaccuracies from poor recall [37, 38]. Having a low share of vaccinations reported from a vaccination card suggests weaker health care systems. Accordingly, countries with a greater share of vaccine information reported by mothers from memory (rather than from a card) or missing vaccine information also had higher zero-dose prevalence, which suggests that recall bias and missingness may not be random. Further, vaccination data from cards may also contain inaccuracies [39]. However, these surveys often provide the only nationally representative data on vaccine coverage available and are an important complement to administrative data. Further, although no “gold-standard” exists regarding vaccine information, excluding caregivers recall would bias vaccine coverage further and is not recommended [37].

Third, the varying times of surveys can complicate the interpretation of the findings. For some countries, the most recent survey was conducted several years ago, and the situation may, in general, have improved. Our focus was, however, on change across time, which was standardized as average annual change. A few surveys, for example, from India, were conducted during the Covid-19 pandemic, which may have disrupted immunization efforts. Fourth, differences between the MICS and DHS should be kept in mind: while DHS only includes biological children of interviewed women, the MICS also includes foster children. Discrepancies across different data sources are a common problem when comparing vaccine coverage, for example, in the WHO and UNICEF estimates of national immunization coverage (WUENIC) [40].

Finally, we coded children with missing vaccine information and mothers who did not know whether child received the vaccine as not having received a vaccine, which may bias the zero-dose prevalence upward. However, for most surveys in our analysis, the information on whether children received the vaccine was close to complete. We further conducted sensitivity analyses, excluding these children (rather than coding them as zero-dose), and found that the link between zero-dose prevalence and postneonatal and child mortality attenuated considerably, suggesting reduced concordance with this composite child health measure.

## Conclusions

Our results indicate impressive improvements in vaccination coverage between 2005 and 2019, with a 0.7% point decline in the percentage of children not receiving any DPT-containing vaccine per year on average in our pooled sample. However, the zero-dose prevalence remains high in some countries. Further, vaccination information was often reported by mothers from memory or in some cases missing, especially in countries with high zero-dose prevalence, which should be kept in mind when interpreting the findings from this study. Efforts to reduce the number of zero-dose children should focus on countries with high prevalence and populous countries with large numbers of non-vaccinated children to achieve the Immunization Agenda 2030.

## Electronic Supplementary Material

Below is the link to the electronic supplementary material.

Supplementary Material 1

## Data Availability

DHS data are available at https://dhsprogram.com and MICS data at https://mics.unicef.org (requiring a simple application).

## Abbreviations

CI: Confidence Interval
DHS: Demographic and Health Surveys
DTP: Diphtheria-Pertussis-Tetanus
MICS: Multiple Indicator Cluster Surveys
WUENIC: WHO and UNICEF estimates of National Immunization Coverage
