# Correction: Prevalence and Trends of Not Receiving a Dose of DPT-Containing Vaccine Among Children 12–35 Months: An Analysis of 81 Low- And Middle-Income Countries

**DOI:** 10.1007/s44197-025-00389-8

**Published:** 2025-04-03

**Authors:** Omar Karlsson, Sunil Rajpal, Mira Johri, Rockli Kim, SV Subramanian

**Affiliations:** 1 Duke University Population Research Institute, Duke University, 140 Science Dr, Durham, NC 27710 USA; 2 Centre for Economic Demography, School of Economics and Management, Lund University, P.O. Box 7083, Lund, 220 07 Sweden; 3 Department of Economics, FLAME University, Pune, India; 4 Carrefour de l’Innovation, Centre de Recherche du Centre Hospitalier de l Universite, de Montréal (CRCHUM), Montréal, QC Canada; 5 Département de Gestion, d’Évaluation, École de Santé Publique, et de Politique de Santé, Université de Montréal (ÉSPUM), Montréal, QC Canada; 6 Division of Health Policy & Management, College of Health Science, Korea University, 145 Anam-ro, Seongbuk-gu, Seoul, 02841 Republic of Korea; 7 Interdisciplinary Program in Precision Public Health, Department of Public Health Sciences, Graduate School of Korea University, 145 Anam-ro, Seongbuk-gu, Seoul, 02841 Republic of Korea; 8 Harvard Center for Population and Development Studies, 9 Bow Street, Cambridge, MA 02138 USA; 9 Department of Social and Behavioral Sciences, Harvard T.H. Chan School of Public Health, Boston, MA 02115 USA

**Correction: Journal of Epidemiology and Global Health (2024) 14:1490–1503** 10.1007/s44197-024-00294-6

After the article was published, the authors identified a coding error that affected the estimates of zero-dose children in the Demographic and Health Surveys (DHS) data. Specifically, children who had died by the time of the survey were erroneously included in the measure of zero-dose children. This issue did not affect estimates derived from the Multiple Indicator Cluster Surveys (MICS). We have now corrected this error and provide updated tables and figures.

Despite these minor numerical adjustments, the conclusions of the study remain unchanged:

- Zero-dose prevalence has declined globally, with Gavi-eligible countries experiencing a significantly faster reduction.
- Reductions in zero-dose prevalence are associated with declines in postneonatal and child mortality, underscoring the importance of immunization efforts.
- Efforts should prioritize countries with high prevalence and number of zero-dose children to achieve Immunization Agenda 2030 targets.

The corrections resulted in minor changes to the estimated prevalence of zero-dose children in most countries. Importantly, all key findings remain robust, including:

- The sharp decline in zero-dose prevalence over time.
- The association between Gavi-eligibility and the change in zero-dose prevalence.
- The relationship between changes in zero-dose prevalence and changes in postneonatal and child mortality.
- The sensitivity analyses, as reported in the original supplement, were unaffected and continue to show similar results as the main analysis.

The overall prevalence in the most recent surveys was 12.4% after the correction (instead of 15.9%), with an average annual decline in prevalence of 0.7 pp (instead of 0.8 pp). The association between the change in postneonatal and child deaths per 1000 births and percentage point change in zero-dose prevalence was 1.2 deaths (instead of 1.4).

While the overall impact of the error was small, a few countries had noteworthy changes in the estimated prevalence of zero-dose children (listed here for the latest surveys):

- Sierra Leone: 9 percentage point (pp) change in prevalence.
- Liberia: 7 pp change.
- Chad, Benin, Nigeria, Burundi, Lesotho, and Haiti: 6 pp change.
- Malawi, Cameroon, Zambia, Mali, Zimbabwe, Guinea, Pakistan: 5 pp change.

Conclusion

The corrected analysis confirms that substantial progress has been made in reducing the percentage of children who have not received a DPT-containing vaccine. The main conclusions of the study remain unchanged:

- The prevalence of zero-dose children has declined globally, with Gavi-eligible countries experiencing a significantly faster reduction.
- A decrease in zero-dose prevalence is associated with a decline in postneonatal and child mortality, reinforcing the importance of immunization efforts.
- Efforts to reduce the number of zero-dose children should focus on countries with high prevalence and large populations of unvaccinated children to meet the Immunization Agenda 2030 targets.

Subsequently, Figs. 1, 2 and 3 and Tables 1, 2, 3 and 4 were corrected.

**Fig. 1:**
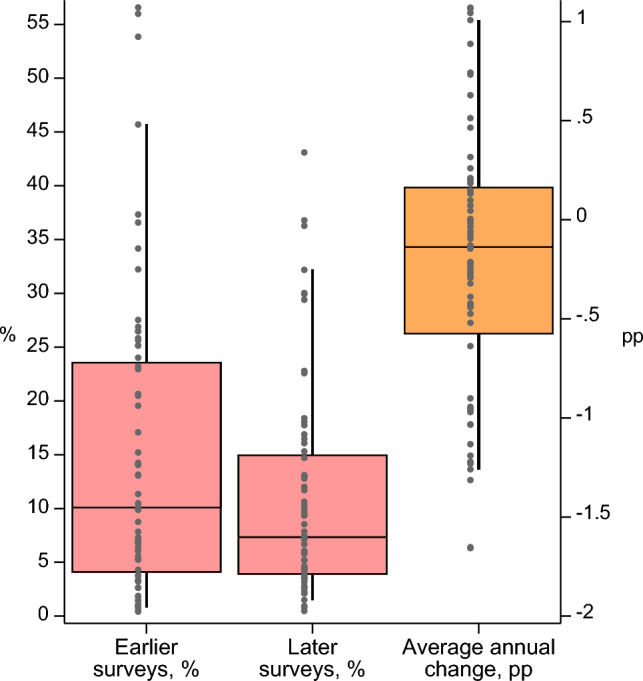
Distribution of zero-dose prevalence across countries by survey year. *Note* Only includes countries with two surveys. Percentiles 5 and 95 (line) and 25, 50, and 75 (box) are shown. Dots indicate country estimates. Each country’s estimate was weighted using sampling weights. Surveys were equally weighted for the median and percentiles. See Table 2 for tabulated estimates. Percentage point (pp) average annual change (AAC) is shown on the right side y-axis

**Fig. 2:**
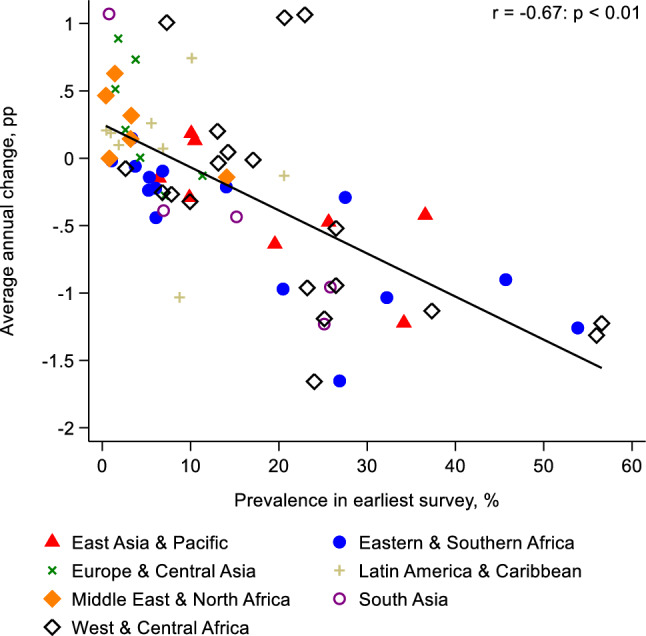
The relationship between country-level zero-dose prevalence in the earliest survey and average annual percentage point (pp) change in prevalence. *Note* Pearson’s correlation coefficient (r) is shown. Countries were equally weighted

**Fig. 3:**
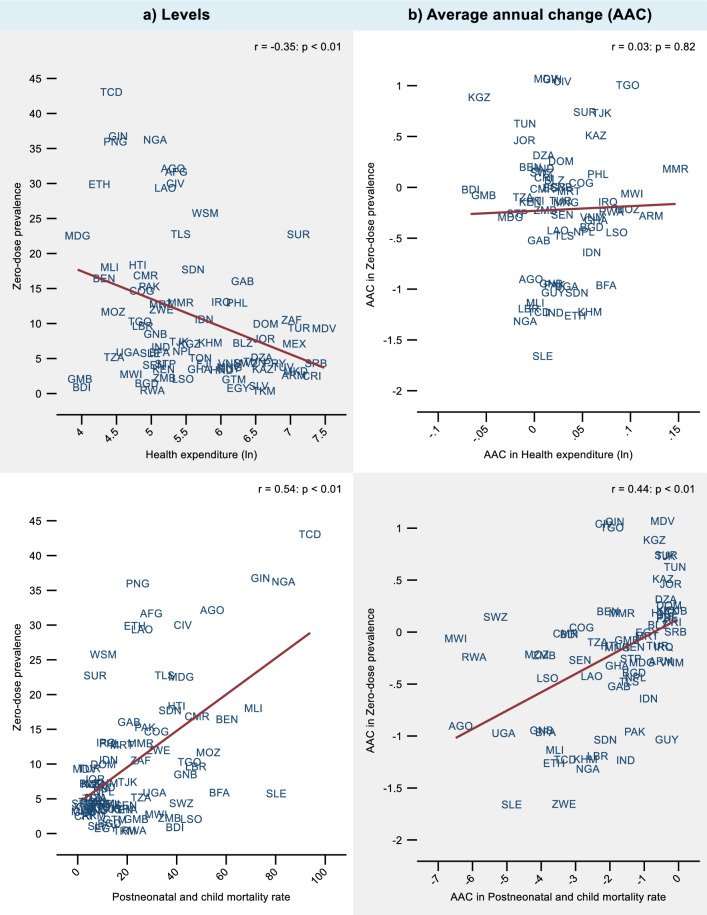
Correlation of zero-dose prevalence with health expenditure and postneonatal and child mortality rate. *Notes* The y and x-axes vary across graphs. GDP and health expenditure were measured per capita in PPP adjusted constant 2017 international $. Pearson’s correlation coefficients (r) are shown. Postneonatal and child mortality rate is deaths per 1000 live births. AAC indicates variables expressed as average annual (absolute) change. Countries were equally weighted

**Table 1:** Zero-dose prevalence and estimated number of zero-dose children

	Survey year	Prevalence (%)	95% confidence interval	Number (thousands)
Pooled	2019*	12.4	12.0, 12.9	21,096
East Asia and Pacific	2019*	11.2	10.4, 12.0	2306
Cambodia	2022	7.3	6.1, 8.6	46
Fiji	2021	4.3	3.0, 6.1	1.5
Indonesia	2017	10.6	9.5, 11.9	991
Lao	2017	29.4	27.3, 31.6	93
Mongolia	2018	3.8	2.8, 5.2	5.9
Myanmar	2016	13.0	10.4, 16.2	234
Papua New Guinea	2018	36.0	32.7, 39.4	170
Philippines	2022	13.0	11.0, 15.3	627
Samoa	2020	25.8	22.2, 29.8	3.0
Timor-Leste	2016	22.8	20.2, 25.6	14
Tonga	2019	5.1	3.1, 8.3	0.25
Tuvalu	2020	3.8	2.0, 7.0	0.019
Viet Nam	2021	4.3	3.1, 6.0	128
Eastern and Southern Africa	2018*	14.5	13.2, 16.0	4682
Angola	2016	32.2	29.2, 35.3	692
Burundi	2017	0.9	0.7, 1.3	7.8
Eswatini	2014	4.4	3.2, 5.9	2.7
Ethiopia	2019	29.9	25.0, 35.3	2051
Kenya	2022	3.5	2.9, 4.2	97
Lesotho	2014	2.1	1.4, 3.1	2.3
Madagascar	2021	22.6	20.2, 25.1	378
Malawi	2016	2.8	2.2, 3.4	32
Mozambique	2015	11.7	9.1, 15.0	221
Rwanda	2020	0.5	0.3, 0.8	3.6
South Africa	2016	10.5	8.4, 13.2	245
Sudan	2014	17.7	15.4, 20.4	430
Tanzania	2022	5.2	4.3, 6.3	227
Uganda	2016	5.9	5.1, 6.8	165
Zambia	2019	2.3	1.7, 3.1	27
Zimbabwe	2015	12.0	10.0, 14.4	111
Europe and Central Asia	2018*	7.2	6.1, 8.6	361
Armenia	2016	2.6	1.6, 4.4	2.3
Kazakhstan	2015	3.5	2.7, 4.6	27
Kosovo	2020	3.9	2.6, 5.9	1.5
Kyrgyzstan	2018	7.1	5.5, 9.2	23
North Macedonia	2019	3.2	1.6, 6.2	1.4
Serbia	2019	4.4	2.7, 7.0	6.1
Tajikistan	2017	7.4	6.0, 9.2	38
Turkmenistan	2016	0.4	0.2, 0.9	1.2
Türkiye	2019	9.4	7.4, 11.8	262
Latin America and Caribbean	2016*	6.8	5.5, 8.4	505
Belize	2016	7.2	5.0, 10.4	1.1
Costa Rica	2018	2.6	1.6, 4.0	3.6
Cuba	2019	3.2	2.0, 4.9	7.3
Dominican Republic	2019	10.0	8.7, 11.5	41
El Salvador	2014	1.1	0.7, 1.8	2.6
Guatemala	2015	2.0	1.6, 2.6	16
Guyana	2014	3.6	2.5, 5.2	1.1
Haiti	2017	18.4	15.8, 21.3	93
Honduras	2019	3.4	2.6, 4.3	14
Mexico	2015	7.1	5.1, 10.0	312
Paraguay	2016	4.3	3.4, 5.6	12
Suriname	2018	22.8	19.5, 26.3	4.9
Middle East and North Africa	2016*	4.9	4.5, 5.4	515
Algeria	2019	5.2	4.4, 6.1	103
Egypt	2014	0.8	0.5, 1.2	40
Iraq	2018	13.1	11.6, 14.7	298
Jordan	2018	7.8	6.3, 9.7	37
State of Palestine	2020	4.6	3.6, 5.9	13
Tunisia	2018	4.6	3.5, 6.0	20
South Asia	2020*	8.6	8.0, 9.2	5718
Afghanistan	2015	31.7	27.9, 35.8	742
Bangladesh	2018	1.5	1.0, 2.3	87
India	2021	6.7	6.4, 7.0	3056
Maldives	2017	9.3	7.0, 12.3	1.4
Nepal	2022	6.1	4.8, 7.6	72
Pakistan	2018	15.3	12.5, 18.7	1789
West and Central Africa	2019*	26.0	24.8, 27.3	7178
Benin	2018	16.5	14.6, 18.6	134
Burkina Faso	2021	5.9	4.8, 7.3	85
Cameroon	2019	16.9	14.5, 19.5	288
Chad	2015	43.1	40.1, 46.1	488
Congo	2015	14.7	12.8, 16.8	49
Cote d’Ivoire	2021	30.0	27.5, 32.7	507
Gabon	2021	16.1	13.7, 18.8	20
Gambia	2020	2.1	1.5, 2.9	3.5
Ghana	2023	3.5	2.7, 4.5	62
Guinea	2018	36.8	33.3, 40.4	301
Guinea-Bissau	2019	8.5	7.1, 10.3	10.0
Liberia	2020	9.7	7.7, 12.1	29
Mali	2018	18.1	15.6, 20.8	273
Mauritania	2021	12.8	11.2, 14.5	36
Nigeria	2018	36.3	34.1, 38.5	4864
Sao Tome and Principe	2019	4.3	3.0, 6.1	0.52
Senegal	2019	4.1	2.9, 5.7	41
Sierra Leone	2019	5.8	4.8, 7.0	27
Togo	2017	10.3	8.5, 12.5	50

*Average survey year is shown for pooled and regional estimates. The number of zero-dose children was estimated using the zero-dose prevalence and an estimate of the population of children 12–35 months old obtained from the United Nation World Population Prospects linked to the country and year of survey. Estimates refer to the latest survey in each country. 95% confidence intervals (CI) were adjusted for clustering at the level of primary sampling units. Estimates were weighted using sampling weights rescaled to sum up the population 12–35 months old in the country and year of survey

**Table 2:** Zero-dose prevalence and average annual percentage point (pp) change in prevalence

	Earlier survey			Later survey			Average annual change	
	Survey year	Zero-dose	95% CI	Survey year	Zero-dose	95% CI	Change	95% CI
		%	%		%	%	pp	%
Pooled	2005**	22.5	21.7, 23.3	2019**	12.3	11.9, 12.8	− 0.7*	− 0.8, − 0.6
East Asia and Pacific	2002**	15.8	14.4, 17.2	2019**	10.6	9.8, 11.5	− 0.3*	− 0.4, − 0.2
Cambodia	2000	34.2	30.9, 37.5	2022	7.3	6.1, 8.6	− 1.2*	− 1.4, − 1.1
Indonesia	2003	19.5	17.0, 22.3	2017	10.6	9.5, 11.9	− 0.6*	− 0.8, − 0.4
Lao	2000	36.6	32.6, 40.7	2017	29.4	27.3, 31.6	− 0.4*	− 0.7, − 0.2
Mongolia	2000	6.4	4.8, 8.5	2018	3.8	2.8, 5.2	− 0.1*	− 0.3, − 0.0
Myanmar	2000	10.1	8.8, 11.6	2016	13.0	10.4, 16.2	0.2	− 0.0, 0.4
Philippines	2003	10.5	9.1, 12.1	2022	13.0	11.0, 15.3	0.1	− 0.0, 0.3
Timor-Leste	2010	25.6	23.1, 28.4	2016	22.8	20.2, 25.6	− 0.5	− 1.1, 0.2
Viet Nam	2002	9.9	6.8, 14.2	2021	4.3	3.1, 6.0	− 0.3*	− 0.5, − 0.1
Eastern and Southern Africa	2003**	24.9	23.5, 26.4	2018**	14.8	13.4, 16.4	− 0.7*	− 0.8, − 0.5
Angola	2001	45.7	42.6, 48.8	2016	32.2	29.2, 35.3	− 0.9*	− 1.2, − 0.6
Burundi	2011	1.1	0.7, 1.5	2017	0.9	0.7, 1.3	− 0.0	− 0.1, 0.1
Eswatini	2007	3.3	2.3, 4.8	2014	4.4	3.2, 5.9	0.1	− 0.1, 0.4
Ethiopia	2000	53.8	50.3, 57.4	2019	29.9	25.0, 35.3	− 1.3*	− 1.6, − 0.9
Kenya	2009	5.3	4.0, 7.0	2022	3.5	2.9, 4.2	− 0.1*	− 0.3, − 0.0
Lesotho	2005	6.1	4.6, 7.9	2014	2.1	1.4, 3.1	− 0.4*	− 0.6, − 0.2
Madagascar	2004	27.5	21.9, 33.9	2021	22.6	20.2, 25.1	− 0.3	− 0.7, 0.1
Malawi	2000	3.7	3.0, 4.6	2016	2.8	2.2, 3.4	− 0.1	− 0.1, 0.0
Mozambique	2004	14.0	11.8, 16.7	2015	11.7	9.1, 15.0	− 0.2	− 0.6, 0.1
Rwanda	2000	5.2	4.3, 6.3	2020	0.5	0.3, 0.8	− 0.2*	− 0.3, − 0.2
Sudan	2000	32.2	30.2, 34.3	2014	17.7	15.4, 20.4	− 1.0*	− 1.3, − 0.8
Tanzania	2005	6.8	5.2, 9.0	2022	5.2	4.3, 6.3	− 0.1	− 0.2, 0.0
Uganda	2001	20.5	17.8, 23.4	2016	5.9	5.1, 6.8	− 1.0*	− 1.2, − 0.8
Zambia	2002	6.0	4.9, 7.5	2019	2.3	1.7, 3.1	− 0.2*	− 0.3, − 0.1
Zimbabwe	2006	26.9	23.8, 30.2	2015	12.0	10.0, 14.4	− 1.7*	− 2.1, − 1.2
Europe and Central Asia	2007**	7.8	6.3, 9.5	2018**	7.7	6.5, 9.2	− 0.0	− 0.2, 0.2
Armenia	2000	7.1	5.0, 9.9	2016	2.6	1.6, 4.4	− 0.3*	− 0.5, − 0.1
Kazakhstan	2011	1.5	0.9, 2.4	2015	3.5	2.7, 4.6	0.5*	0.2, 0.8
Kosovo	2014	2.6	1.5, 4.5	2020	3.9	2.6, 5.9	0.2	− 0.1, 0.6
Kyrgyzstan	2012	1.8	1.1, 2.9	2018	7.1	5.5, 9.2	0.9*	0.5, 1.2
Serbia	2006	4.3	3.2, 5.7	2019	4.4	2.7, 7.0	0.0	− 0.2, 0.2
Tajikistan	2012	3.8	2.8, 5.0	2017	7.4	6.0, 9.2	0.7*	0.3, 1.1
Türkiye	2004	11.3	9.0, 14.2	2019	9.4	7.4, 11.8	− 0.1	− 0.4, 0.1
Latin America and Caribbean	2004**	7.2	6.0, 8.6	2018**	9.2	8.3, 10.2	0.1*	0.0, 0.2
Belize	2011	6.9	5.1, 9.3	2016	7.2	5.0, 10.4	0.1	− 0.6, 0.8
Costa Rica	2011	1.9	0.9, 3.7	2018	2.6	1.6, 4.0	0.1	− 0.2, 0.3
Cuba	2006	0.5	0.2, 0.9	2019	3.2	2.0, 4.9	0.2*	0.1, 0.3
Dominican Republic	2002	5.6	4.7, 6.6	2019	10.0	8.7, 11.5	0.3*	0.2, 0.4
Guyana	2009	8.8	6.7, 11.3	2014	3.6	2.5, 5.2	− 1.0*	− 1.6, − 0.5
Haiti	2000	20.6	16.7, 25.1	2017	18.4	15.8, 21.3	− 0.1	− 0.4, 0.2
Honduras	2006	1.0	0.6, 1.4	2019	3.4	2.6, 4.3	0.2*	0.1, 0.3
Suriname	2001	10.1	7.7, 13.3	2018	22.8	19.5, 26.3	0.7*	0.5, 1.0
Middle East and North Africa	2006**	4.7	4.3, 5.1	2016**	4.9	4.5, 5.4	0.0	− 0.0, 0.1
Algeria	2013	3.3	2.7, 4.0	2019	5.2	4.4, 6.1	0.3*	0.1, 0.5
Egypt	2000	0.8	0.5, 1.2	2014	0.8	0.5, 1.2	− 0.0	− 0.0, 0.0
Iraq	2011	14.1	13.0, 15.2	2018	13.1	11.6, 14.7	− 0.1	− 0.4, 0.1
Jordan	2002	0.4	0.2, 0.8	2018	7.8	6.3, 9.7	0.5*	0.4, 0.6
State of Palestine	2010	3.2	2.6, 3.9	2020	4.6	3.6, 5.9	0.1*	0.0, 0.3
Tunisia	2013	1.4	0.9, 2.4	2018	4.6	3.5, 6.0	0.6*	0.3, 0.9
South Asia	2006**	23.3	22.1, 24.5	2020**	7.7	7.1, 8.4	− 1.1*	− 1.2, − 1.0
Bangladesh	2004	6.9	5.4, 8.9	2018	1.5	1.0, 2.3	− 0.4*	− 0.5, − 0.3
India	2006	25.1	23.7, 26.6	2021	6.7	6.4, 7.0	− 1.2*	− 1.3, − 1.1
Maldives	2009	0.8	0.4, 1.5	2017	9.3	7.0, 12.3	1.1*	0.7, 1.4
Nepal	2001	15.2	12.1, 18.9	2022	6.1	4.8, 7.6	− 0.4*	− 0.6, − 0.3
Pakistan	2007	25.8	23.5, 28.3	2018	15.3	12.5, 18.7	− 1.0*	− 1.3, − 0.6
West and Central Africa	2004**	37.2	34.3, 40.2	2019**	26.0	24.8, 27.3	− 0.8*	− 1.0, − 0.6
Benin	2001	13.1	10.6, 15.9	2018	16.5	14.6, 18.6	0.2*	0.0, 0.4
Burkina Faso	2003	23.2	19.9, 26.9	2021	5.9	4.8, 7.3	− 1.0*	− 1.2, − 0.8
Cameroon	2004	17.1	14.8, 19.7	2019	16.9	14.5, 19.5	− 0.0	− 0.2, 0.2
Chad	2004	56.6	50.7, 62.2	2015	43.1	40.1, 46.1	− 1.2*	− 1.8, − 0.6
Congo	2005	14.2	11.4, 17.6	2015	14.7	12.8, 16.8	0.0	− 0.3, 0.4
Cote d’Ivoire	2012	20.6	17.8, 23.8	2021	30.0	27.5, 32.7	1.0*	0.6, 1.5
Gabon	2001	26.5	23.6, 29.5	2021	16.1	13.7, 18.8	− 0.5*	− 0.7, − 0.3
Gambia	2013	2.6	1.9, 3.6	2020	2.1	1.5, 2.9	− 0.1	− 0.2, 0.1
Ghana	2003	9.9	8.0, 12.3	2023	3.5	2.7, 4.5	− 0.3*	− 0.4, − 0.2
Guinea	2005	22.9	20.0, 26.2	2018	36.8	33.3, 40.4	1.1*	0.7, 1.4
Guinea-Bissau	2000	26.5	26.5, 26.5	2019	8.5	7.1, 10.3	− 0.9*	− 1.0, − 0.9
Liberia	2007	25.2	20.2, 30.8	2020	9.7	7.7, 12.1	− 1.2*	− 1.6, − 0.7
Mali	2001	37.3	34.3, 40.5	2018	18.1	15.6, 20.8	− 1.1*	− 1.4, − 0.9
Mauritania	2011	13.1	11.5, 15.0	2021	12.8	11.2, 14.5	− 0.0	− 0.3, 0.2
Nigeria	2003	56.0	50.8, 61.0	2018	36.3	34.1, 38.5	− 1.3*	− 1.7, − 0.9
Sao Tome and Principe	2009	6.8	4.9, 9.4	2019	4.3	3.0, 6.1	− 0.3	− 0.5, 0.0
Senegal	2005	7.8	6.7, 9.2	2019	4.1	2.9, 5.7	− 0.3*	− 0.4, − 0.1
Sierra Leone	2008	24.0	21.2, 27.1	2019	5.8	4.8, 7.0	− 1.7*	− 1.9, − 1.4
Togo	2014	7.3	5.8, 9.2	2017	10.3	8.5, 12.5	1.0*	0.1, 1.9

*p < 0.05. **Average survey year is shown for pooled and regional estimates. 95% confidence intervals (CI) were adjusted for clustering at the level of primary sampling units. Estimates were weighted using sampling weights rescaled to sum up the population 12–35 months old in the country and year of survey

**Table 3:** Linear regressions

	(1)	(2)	(3)	(4)
	Level in latest survey		Average annual change	
	Zero-dose prevalence	Postneonatal and child mortality	Zero-dose prevalence	Postneonatal and child mortality
Zero-dose prevalence		0.59*		1.23*
		(0.24)		(0.27)
Total health expenditure (ln)	− 8.10*	− 1.62	2.02	− 7.42
	(2.82)	(4.53)	(2.85)	(7.68)
GDP (ln)	7.57*	− 7.10	− 0.34	9.92
	(2.77)	(4.71)	(6)	(12.27)
Population below age 5 (%)	− 0.24*	− 0.50*	0.06	− 0.15
	(0.09)	(0.13)	(0.03)	(0.09)
Survey year	− 0.04	− 0.77	− 0	0.12
	(0.41)	(0.52)	(0.03)	(0.09)
Gavi-eligible			− 0.57*	
			(0.14)	
Constant	11.05*	25.52*	− 0.22*	− 2.03*
	(0.99)	(1.42)	(0.08)	(0.19)
R squared	0.30	0.67	0.19	0.26
Observations	78	78	64	64

*P < 0.05. Dependent variables are indicated at the top of each column. In columns 3 and 4, all variables are expressed as average annual absolute change, except for survey year and Gavi-eligibility. All values were mean-centered. GDP and health expenditure are per capita in PPP adjusted constant 2017 international $. Postneonatal and child mortality rate is expressed as deaths per 1000 live births. Countries were equally weighted. Robust standard errors are shown in parentheses below coefficients

**Table 4:** Difference between previously published estimates and corrected estimates of zero-dose prevalence in the latest DHS surveys (from Table 1)

	Published	Corrected	Difference
	%	%	pp
Pooled	16	12	3.5
East Asia and Pacific	13	11	1.9
Cambodia	8.2	7.3	0.94
Indonesia	13	11	2.1
Myanmar	16	13	3.1
Papua New Guinea	39	36	3.4
Philippines	15	13	2.3
Timor-Leste	26	23	2.9
Eastern and Southern Africa	18	15	3.5
Angola	35	32	3.3
Burundi	6.9	0.93	5.9
Ethiopia	33	30	3.4
Kenya	6.9	3.5	3.4
Lesotho	8.3	2.1	6.2
Madagascar	27	23	4.3
Malawi	7.6	2.8	4.8
Mozambique	15	12	3.5
Rwanda	3.9	0.48	3.5
South Africa	14	11	3.0
Tanzania	8.5	5.2	3.3
Uganda	10	5.9	4.4
Zambia	7.2	2.3	4.9
Zimbabwe	17	12	5.1
Europe and Central Asia	8.5	7.2	1.3
Armenia	3.0	2.6	0.37
Tajikistan	10	7.4	2.8
Türkiye	11	9.4	1.7
Latin America and Caribbean	7.6	6.8	0.79
Guatemala	5.1	2.0	3.0
Haiti	25	18	6.3
Middle East and North Africa	6.1	4.9	1.2
Egypt	3.1	0.79	2.3
Jordan	9.6	7.8	1.8
South Asia	12	8.6	3.8
Afghanistan	35	32	3.4
Bangladesh	5.2	1.5	3.8
India	10	6.7	3.4
Maldives	11	9.3	1.3
Nepal	8.5	6.1	2.4
Pakistan	21	15	5.4
West and Central Africa	31	26	5.3
Benin	22	16	5.8
Burkina Faso	9.1	5.9	3.2
Cameroon	22	17	4.9
Chad	49	43	5.7
Cote d’Ivoire	34	30	4.3
Gabon	19	16	2.9
Gambia	6.5	2.1	4.4
Ghana	6.1	3.5	2.6
Guinea	42	37	5.2
Liberia	17	9.7	7.3
Mali	23	18	5.0
Mauritania	16	13	3.4
Nigeria	42	36	5.9
Senegal	7.4	4.1	3.3
Sierra Leone	15	5.8	9.0

A correction was only done for estimates from DHS surveys. The overall Pearson’s correlation between the published and corrected estimates was over 0.98. A coding error caused estimates to include children who had died by the time of the survey. The corrected estimates exclude these children. Pooled estimates and estimates for global regions used all surveys (i.e., both MICS and DHS). Estimates were weighted using sampling weights rescaled to sum up the population 12–35 months old in the country and year of survey

The Original Article has been updated.

