# Supplementary material for: Prevalence and Trends of Not Receiving a Dose of DPT-Containing Vaccine Among Children 12–35 Months: An Analysis of 81 Low- And Middle-Income Countries
Source: J Epidemiol Glob Health. 2024 Sep 19;14(4):1490–503. doi: 10.1007/s44197-024-00294-6 (PMC11652464; doi:10.1007/s44197-024-00294-6)
Supplement: Supplementary file 1 — Supplementary Material 1 [file 44197_2024_294_MOESM1_ESM.docx]

**Supplement**

**Prevalence and trends of not receiving a dose of DPT-containing vaccine among children 12–35 months: An analysis of 81 low- and middle-income countries**

[Table S1. Survey year, data source, sample size, and Gavi-eligibility 2](#_Toc171262132)

[Table S2. Tabulated estimates from Figure 1: Distribution of zero-dose prevalence across countries 4](#_Toc171262133)

[Table S3. Missing DPT1 information and vaccination reported by mother or on card 5](#_Toc171262134)

[Sensitivity analysis: Excluding children with missing DPT vaccine information (instead of coding them as zero-dose) 8](#_Toc171262135)

[Figure S1. Distribution of zero-dose prevalence across countries by survey year: excluding children with missing DPT vaccine information (instead of coding them as zero-dose) 9](#_Toc171262136)

[Table S4. Tabulated estimates from Figure 1: Distribution of zero-dose prevalence across countries: excluding children with missing DPT vaccine information (instead of coding them as zero-dose) 10](#_Toc171262137)

[Table S5. Zero-dose prevalence and estimated number of zero-dose children: excluding children with missing DPT vaccine information (instead of coding them as zero-dose) 11](#_Toc171262138)

[Table S6. Zero-dose prevalence and average annual percentage point (pp) change in prevalence: excluding children with missing DPT vaccine information (instead of coding them as zero-dose) 13](#_Toc171262139)

[Figure S2. The relationship between country-level zero-dose prevalence in the earliest survey and average annual percentage point (pp) change in prevalence: excluding children with missing DPT vaccine information (instead of coding them as zero-dose) 15](#_Toc171262140)

[Figure S3. Correlation of zero-dose prevalence with health expenditure and postneonatal and child mortality rate: excluding children with missing DPT vaccine information (instead of coding them as zero-dose) 16](#_Toc171262141)

[Table S7. Linear regressions: excluding children with missing DPT vaccine information (instead of coding them as zero-dose) 17](#_Toc171262142)

[Sensitivity analysis: Excluding children with missing DPT vaccine information and whose mothers answered that they did not know whether the child was vaccinated with DPT (instead of coding them as zero-dose) 18](#_Toc171262143)

[Figure S4. Distribution of zero-dose prevalence across countries by survey year: excluding children with missing DPT vaccine information and whose mothers answered that they did not know whether the child was vaccinated with DPT (instead of coding them as zero-dose) 19](#_Toc171262144)

[Table S8. Tabulated estimates from Figure 1: Distribution of zero-dose prevalence across countries: excluding children with missing DPT vaccine information and whose mothers answered that they did not know whether the child was vaccinated with DPT (instead of coding them as zero-dose) 20](#_Toc171262145)

[Table S9. Zero-dose prevalence and estimated number of zero-dose children: excluding children with missing DPT vaccine information and whose mothers answered that they did not know whether the child was vaccinated with DPT (instead of coding them as zero-dose) 21](#_Toc171262146)

[Table S10. Zero-dose prevalence and average annual percentage point (pp) change in prevalence: excluding children with missing DPT vaccine information and whose mothers answered that they did not know whether the child was vaccinated with DPT (instead of coding them as zero-dose) 23](#_Toc171262147)

[Figure S5. The relationship between country-level zero-dose prevalence in the earliest survey and average annual percentage point (pp) change in prevalence: excluding children with missing DPT vaccine information and whose mothers answered that they did not know whether the child was vaccinated with DPT (instead of coding them as zero-dose) 25](#_Toc171262148)

[Figure S6. Correlation of zero-dose prevalence with health expenditure and postneonatal and child mortality rate: excluding children with missing DPT vaccine information and whose mothers answered that they did not know whether the child was vaccinated with DPT (instead of coding them as zero-dose) 26](#_Toc171262149)

[Table S11. Linear regressions: excluding children with missing DPT vaccine information and whose mothers answered that they did not know whether the child was vaccinated with DPT (instead of coding them as zero-dose) 28](#_Toc171262150)

## Table S1. Survey year, data source, sample size, and Gavi-eligibility

|  | Earlier surveys | | | | Later surveys | | | |
| --- | --- | --- | --- | --- | --- | --- | --- | --- |
|  | Survey | Data | Full | Gavi- | Survey | Data | Full | Gavi- |
|  | year | source | sample | eligible | year | source | sample | eligible |
|  |  |  |  |  |  |  |  |  |
| Pooled |  |  | 207,687 |  |  |  | 345,660 |  |
| Afghanistan |  |  |  |  | 2015 | DHS | 12,537 | Yes |
| Algeria | 2012–13 | MICS | 5,949 | No | 2018–19 | MICS | 5,881 | No |
| Angola | 2001 | MICS | 2,272 | Yes | 2015–16 | DHS | 5,534 | Yes |
| Armenia | 2000 | DHS | 598 | Yes | 2015–16 | DHS | 683 | No |
| Bangladesh | 2004 | DHS | 2,571 | Yes | 2017–18 | DHS | 3,315 | Yes |
| Belize | 2011 | MICS | 801 | No | 2015–16 | MICS | 1,029 | No |
| Benin | 2001 | DHS | 1,869 | Yes | 2017–18 | DHS | 4,886 | Yes |
| Burkina Faso | 2003 | DHS | 3,566 | Yes | 2021 | DHS | 4,350 | Yes |
| Burundi | 2010–11 | DHS | 3,013 | Yes | 2016–17 | DHS | 5,022 | Yes |
| Cambodia | 2000 | DHS | 2,813 | Yes | 2021–22 | DHS | 3,186 | Yes |
| Cameroon | 2004 | DHS | 2,933 | Yes | 2018–19 | DHS | 3,586 | Yes |
| Chad | 2004 | DHS | 1,856 | Yes | 2014–15 | DHS | 6,200 | Yes |
| Congo | 2005 | DHS | 1,820 | Yes | 2014–15 | MICS | 3,603 | Yes |
| Costa Rica | 2011 | MICS | 909 | No | 2018 | MICS | 1,469 | No |
| Cote d'Ivoire | 2011–12 | DHS | 2,841 | Yes | 2021 | DHS | 3,770 | Yes |
| Cuba | 2006 | MICS | 1,835 | Yes | 2019 | MICS | 2,150 | Yes |
| Dominican Republic | 2002 | DHS | 4,544 | No | 2019 | MICS | 3,340 | No |
| Egypt | 2000 | DHS | 4,408 | No | 2014 | DHS | 6,409 | No |
| El Salvador |  |  |  |  | 2014 | MICS | 3,014 | No |
| Eswatini | 2006–07 | DHS | 1,015 | No | 2014 | MICS | 1,118 | No |
| Ethiopia | 2000 | DHS | 3,731 | Yes | 2019 | DHS | 2,107 | Yes |
| Fiji |  |  |  |  | 2021 | MICS | 827 | No |
| Gabon | 2000–01 | DHS | 1,674 | No | 2019–21 | DHS | 2,458 | No |
| Gambia | 2013 | DHS | 3,133 | Yes | 2019–20 | DHS | 3,093 | Yes |
| Ghana | 2003 | DHS | 1,407 | Yes | 2022–23 | DHS | 3,677 | Yes |
| Guatemala |  |  |  |  | 2014–15 | DHS | 4,856 | No |
| Guinea | 2005 | DHS | 2,228 | Yes | 2018 | DHS | 2,688 | Yes |
| Guinea-Bissau | 2000 | MICS | 2,247 | Yes | 2018–19 | MICS | 2,881 | Yes |
| Guyana | 2009 | DHS | 900 | Yes | 2014 | MICS | 1,372 | Yes |
| Haiti | 2000 | DHS | 2,499 | Yes | 2016–17 | DHS | 2,439 | Yes |
| Honduras | 2005–06 | DHS | 4,173 | Yes | 2019 | MICS | 3,274 | Yes |
| India | 2005–06 | DHS | 19,296 | Yes | 2019–21 | DHS | 87,730 | Yes |
| Indonesia | 2002–03 | DHS | 6,435 | Yes | 2017 | DHS | 6,942 | Yes |
| Iraq | 2011 | MICS | 14,765 | No | 2018 | MICS | 6,347 | No |
| Jordan | 2002 | DHS | 2,403 | No | 2017–18 | DHS | 4,023 | No |
| Kazakhstan | 2010–11 | MICS | 2,139 | No | 2015 | MICS | 2,196 | No |
| Kenya | 2008–09 | DHS | 2,266 | Yes | 2022 | DHS | 7,342 | Yes |
| Kosovo | 2013–14 | MICS | 646 | No | 2019–20 | MICS | 596 | No |
| Kyrgyzstan | 2012 | DHS | 1,717 | Yes | 2018 | MICS | 1,361 | Yes |
| Lao | 2000 | MICS | 2,057 | Yes | 2017 | MICS | 4,598 | Yes |
| Lesotho | 2004–05 | DHS | 1,346 | Yes | 2014 | DHS | 1,228 | Yes |
| Liberia | 2006–07 | DHS | 2,088 | Yes | 2019–20 | DHS | 2,035 | Yes |
| Madagascar | 2003–04 | DHS | 1,983 | Yes | 2021 | DHS | 4,668 | Yes |
| Malawi | 2000 | DHS | 4,262 | Yes | 2015–16 | DHS | 6,523 | Yes |
| Maldives | 2009 | DHS | 1,551 | No | 2016–17 | DHS | 1,178 | No |
| Mali | 2001 | DHS | 4,253 | Yes | 2018 | DHS | 3,712 | Yes |
| Mauritania | 2011 | MICS | 3,581 | Yes | 2019–21 | DHS | 4,325 | Yes |
| Mexico |  |  |  |  | 2015 | MICS | 3,208 | No |
| Mongolia | 2000 | MICS | 2,448 | Yes | 2018 | MICS | 2,301 | Yes |
| Mozambique | 2003–04 | DHS | 3,522 | Yes | 2015 | DHS | 2,034 | Yes |
| Myanmar | 2000 | MICS | 5,664 | Yes | 2015–16 | DHS | 1,815 | Yes |
| Nepal | 2000–01 | DHS | 2,536 | Yes | 2021–22 | DHS | 2,101 | Yes |
| Nigeria | 2003 | DHS | 1,995 | Yes | 2018 | DHS | 11,953 | Yes |
| North Macedonia |  |  |  |  | 2018–19 | MICS | 598 | No |
| Pakistan | 2006–07 | DHS | 3,220 | Yes | 2017–18 | DHS | 4,716 | Yes |
| Papua New Guinea |  |  |  |  | 2016–18 | DHS | 3,703 | Yes |
| Paraguay |  |  |  |  | 2016 | MICS | 1,939 | No |
| Philippines | 2003 | DHS | 2,738 | No | 2022 | DHS | 3,211 | No |
| Rwanda | 2000 | DHS | 2,512 | Yes | 2019–20 | DHS | 3,164 | Yes |
| Samoa |  |  |  |  | 2019–20 | MICS | 1,119 | No |
| Sao Tome and Principe | 2008–09 | DHS | 755 | No | 2019 | MICS | 754 | No |
| Senegal | 2005 | DHS | 4,061 | Yes | 2019 | DHS | 2,397 | Yes |
| Serbia | 2005–06 | MICS | 1,582 | No | 2019 | MICS | 796 | No |
| Sierra Leone | 2008 | DHS | 1,940 | Yes | 2019 | DHS | 3,606 | Yes |
| South Africa |  |  |  |  | 2016 | DHS | 1,342 | No |
| State of Palestine | 2010 | MICS | 4,385 | No | 2019–20 | MICS | 2,625 | No |
| Sudan | 2000 | MICS | 8,587 | Yes | 2014 | MICS | 5,288 | Yes |
| Suriname | 2000–01 | MICS | 804 | No | 2018 | MICS | 1,699 | No |
| Tajikistan | 2012 | DHS | 2,125 | Yes | 2017 | DHS | 2,523 | Yes |
| Tanzania | 2004–05 | DHS | 3,229 | Yes | 2022 | DHS | 4,183 | Yes |
| Timor-Leste | 2009–10 | DHS | 3,746 | Yes | 2016 | DHS | 2,776 | Yes |
| Togo | 2013–14 | DHS | 2,678 | Yes | 2017 | MICS | 1,961 | Yes |
| Tonga |  |  |  |  | 2019 | MICS | 527 | No |
| Tunisia | 2012–13 | MICS | 1,128 | No | 2018 | MICS | 1,327 | No |
| Turkmenistan |  |  |  |  | 2015–16 | MICS | 1,523 | No |
| Tuvalu |  |  |  |  | 2019–20 | MICS | 213 | No |
| Türkiye | 2003–04 | DHS | 1,705 | No | 2018–19 | DHS | 1,037 | No |
| Uganda | 2000–01 | DHS | 2,589 | Yes | 2016 | DHS | 5,845 | Yes |
| Viet Nam | 2002 | DHS | 915 | Yes | 2020–21 | MICS | 1,687 | Yes |
| Zambia | 2001–02 | DHS | 2,504 | Yes | 2018–19 | DHS | 3,821 | Yes |
| Zimbabwe | 2005–06 | DHS | 1,926 | Yes | 2015 | DHS | 2,310 | Yes |

Notes: Full sample refers to the full sample of children 12–35 months old.

## Table S2. Tabulated estimates from Figure 1: Distribution of zero-dose prevalence across countries

|  | Earlier surveys (%) | Later surveys (%) | AAC (pp) |
| --- | --- | --- | --- |
|  |  |  |  |
| Percentile 5 | 0.8 | 1.5 | -1.259 |
| Percentile 25 | 4.0 | 3.8 | -0.579 |
| Median | 10.0 | 7.3 | -0.141 |
| Percentile 75 | 23.6 | 15.0 | 0.166 |
| Percentile 95 | 45.7 | 32.2 | 1.007 |
| Interquartile range | 19.6 | 11.2 | 0.744 |
| Mean | 14.8 | 10.8 | -0.221 |

Notes: Only countries with two surveys were included. Each country's estimate was weighted using sampling weights. Surveys were equally weighted for the median and percentiles. Percentage point (pp) average annual change (AAC) is shown.

## Table S3. Missing DPT1 information and vaccination reported by mother or on card

|  |  | Missing DPT1 | | Mother does not | | No DPT1 | | DPT1 given, | | DPT1 given, | |
| --- | --- | --- | --- | --- | --- | --- | --- | --- | --- | --- | --- |
|  | Survey | information | | know if got DPT1 | | given | | mother's recall | | on card | |
|  | year | (%) | 95% CI | (%) | 95% CI | (%) | 95% CI | (%) | 95% CI | (%) | 95% CI |
|  |  |  |  |  |  |  |  |  |  |  |  |
| Pooled | 2019* | 0.5 | 0.4, 0.6 | 0.7 | 0.6, 0.7 | 11.4 | 11.0, 11.9 | 23.4 | 23.0, 23.8 | 64.0 | 63.5, 64.6 |
|  |  |  |  |  |  |  |  |  |  |  |  |
| East Asia & Pacific | 2019* | 0.3 | 0.2, 0.3 | 0.5 | 0.4, 0.6 | 10.5 | 9.7, 11.3 | 30.7 | 29.6, 31.9 | 58.0 | 56.7, 59.4 |
| Cambodia | 2022 | 0.0 | na | 0.7 | 0.5, 1.1 | 6.6 | 5.5, 7.8 | 17.4 | 15.2, 19.8 | 75.3 | 72.7, 77.7 |
| Fiji | 2021 | 3.2 | 2.1, 4.8 | 0.5 | 0.2, 1.2 | 0.7 | 0.3, 1.6 | 4.1 | 2.9, 5.8 | 91.6 | 89.2, 93.5 |
| Indonesia | 2017 | 0.1 | 0.0, 0.2 | 0.4 | 0.3, 0.6 | 10.1 | 9.0, 11.4 | 41.3 | 39.7, 43.0 | 48.1 | 46.3, 49.8 |
| Lao | 2017 | 12.9 | 11.4, 14.6 | 2.8 | 2.2, 3.6 | 13.7 | 12.3, 15.1 | 29.5 | 27.7, 31.3 | 41.1 | 38.9, 43.4 |
| Mongolia | 2018 | 1.7 | 1.1, 2.4 | 0.3 | 0.1, 1.1 | 1.8 | 1.1, 3.0 | 8.8 | 7.1, 10.8 | 87.4 | 85.1, 89.4 |
| Myanmar | 2016 | 0.0 | na | 0.5 | 0.2, 1.2 | 12.5 | 9.9, 15.6 | 47.5 | 44.1, 51.1 | 39.4 | 35.6, 43.4 |
| Papua New Guinea | 2018 | 0.1 | 0.0, 0.4 | 2.4 | 1.7, 3.4 | 33.5 | 30.3, 36.9 | 16.5 | 14.7, 18.5 | 47.5 | 44.6, 50.4 |
| Philippines | 2022 | 0.0 | na | 0.5 | 0.3, 1.0 | 12.5 | 10.5, 14.8 | 25.5 | 22.9, 28.2 | 61.5 | 58.5, 64.5 |
| Samoa | 2020 | 14.3 | 12.0, 17.0 | 1.0 | 0.5, 2.0 | 10.5 | 8.2, 13.5 | 22.8 | 20.0, 25.9 | 51.4 | 47.1, 55.7 |
| Timor-Leste | 2016 | 0.0 | na | 0.3 | 0.2, 0.7 | 22.4 | 19.9, 25.2 | 31.2 | 28.9, 33.6 | 46.0 | 43.2, 48.8 |
| Tonga | 2019 | 0.5 | 0.1, 3.6 | 0.0 | na | 4.5 | 2.6, 7.7 | 3.1 | 1.7, 5.5 | 91.8 | 88.5, 94.3 |
| Tuvalu | 2020 | 1.8 | 0.7, 4.6 | 1.4 | 0.5, 4.2 | 0.5 | 0.1, 3.6 | 51.9 | 44.8, 59.0 | 44.3 | 37.5, 51.3 |
| Viet Nam | 2021 | 0.0 | na | 0.0 | na | 4.3 | 3.1, 6.0 | 2.4 | 1.5, 3.8 | 93.3 | 91.2, 94.9 |
|  |  |  |  |  |  |  |  |  |  |  |  |
| Eastern & Southern Africa | 2018* | 0.9 | 0.8, 1.1 | 1.3 | 1.1, 1.5 | 12.3 | 11.0, 13.8 | 24.7 | 23.7, 25.7 | 60.7 | 59.3, 62.2 |
| Angola | 2016 | 0.0 | na | 2.0 | 1.4, 2.9 | 30.2 | 27.2, 33.3 | 31.6 | 29.5, 33.8 | 36.2 | 33.6, 38.9 |
| Burundi | 2017 | 0.0 | na | 0.0 | 0.0, 0.1 | 1.0 | 0.7, 1.3 | 27.4 | 25.6, 29.3 | 71.6 | 69.7, 73.4 |
| Eswatini | 2014 | 2.5 | 1.6, 3.8 | 0.8 | 0.4, 1.7 | 1.1 | 0.6, 1.9 | 11.4 | 8.8, 14.6 | 84.3 | 81.1, 87.0 |
| Ethiopia | 2019 | 0.0 | na | 1.6 | 0.9, 2.8 | 28.4 | 23.8, 33.6 | 16.7 | 13.4, 20.6 | 53.3 | 47.4, 59.0 |
| Kenya | 2022 | 0.0 | na | 0.3 | 0.2, 0.6 | 3.2 | 2.6, 3.9 | 28.1 | 26.5, 29.7 | 68.4 | 66.7, 70.1 |
| Lesotho | 2014 | 0.0 | na | 0.6 | 0.3, 1.2 | 1.6 | 1.0, 2.5 | 24.1 | 21.1, 27.5 | 73.8 | 70.4, 76.9 |
| Madagascar | 2021 | 0.0 | na | 0.3 | 0.1, 0.5 | 22.3 | 19.9, 24.9 | 28.4 | 26.3, 30.7 | 49.0 | 46.2, 51.8 |
| Malawi | 2016 | 0.0 | na | 0.2 | 0.1, 0.4 | 2.6 | 2.1, 3.3 | 27.5 | 26.0, 29.0 | 69.8 | 68.2, 71.3 |
| Mozambique | 2015 | 0.0 | 0.0, 0.3 | 3.5 | 2.4, 5.1 | 8.2 | 5.8, 11.4 | 21.8 | 19.2, 24.8 | 66.5 | 62.8, 69.9 |
| Rwanda | 2020 | 0.0 | na | 0.0 | na | 0.5 | 0.3, 0.8 | 4.7 | 3.8, 5.7 | 94.9 | 93.8, 95.8 |
| South Africa | 2016 | 0.0 | na | 4.5 | 3.3, 6.1 | 6.0 | 4.3, 8.2 | 26.6 | 23.4, 30.0 | 62.9 | 59.1, 66.4 |
| Sudan | 2014 | 12.4 | 10.6, 14.5 | 1.4 | 1.0, 1.9 | 3.9 | 3.1, 4.9 | 47.4 | 44.7, 50.1 | 34.9 | 31.9, 37.9 |
| Tanzania | 2022 | 0.0 | na | 0.5 | 0.3, 0.8 | 4.8 | 3.8, 5.9 | 18.0 | 16.3, 19.9 | 76.8 | 74.8, 78.6 |
| Uganda | 2016 | 0.0 | na | 0.5 | 0.3, 0.8 | 5.4 | 4.7, 6.3 | 31.2 | 29.6, 32.9 | 62.8 | 61.0, 64.7 |
| Zambia | 2019 | 0.0 | na | 0.4 | 0.2, 0.9 | 1.8 | 1.3, 2.6 | 26.6 | 24.7, 28.6 | 71.1 | 69.0, 73.1 |
| Zimbabwe | 2015 | 0.0 | na | 0.1 | 0.0, 0.4 | 11.9 | 9.9, 14.3 | 12.7 | 11.0, 14.6 | 75.3 | 72.8, 77.6 |
|  |  |  |  |  |  |  |  |  |  |  |  |
| Europe & Central Asia | 2018* | 0.2 | 0.1, 0.3 | 4.1 | 3.1, 5.4 | 2.9 | 2.4, 3.6 | 20.3 | 18.3, 22.4 | 72.5 | 70.0, 74.8 |
| Armenia | 2016 | 0.0 | na | 0.0 | na | 1.8 | 1.0, 3.0 | 6.2 | 4.2, 9.0 | 92.1 | 89.1, 94.3 |
| Kazakhstan | 2015 | 1.0 | 0.6, 1.5 | 0.1 | 0.0, 0.3 | 2.5 | 1.8, 3.4 | 2.2 | 1.5, 3.2 | 94.3 | 92.9, 95.5 |
| Kosovo | 2020 | 0.5 | 0.2, 1.6 | 0.6 | 0.2, 1.6 | 2.8 | 1.6, 4.6 | 1.2 | 0.5, 2.5 | 95.0 | 92.6, 96.6 |
| Kyrgyzstan | 2018 | 0.0 | na | 0.0 | na | 7.1 | 5.5, 9.2 | 8.5 | 6.8, 10.6 | 84.4 | 81.4, 87.0 |
| North Macedonia | 2019 | 0.9 | 0.3, 3.0 | 0.0 | na | 2.3 | 1.0, 5.3 | 0.2 | 0.0, 0.9 | 96.6 | 93.6, 98.2 |
| Serbia | 2019 | 1.1 | 0.4, 3.3 | 2.0 | 1.1, 3.8 | 1.2 | 0.6, 2.7 | 13.2 | 10.1, 17.0 | 82.5 | 78.1, 86.1 |
| Tajikistan | 2017 | 0.0 | na | 1.2 | 0.8, 1.7 | 6.5 | 5.1, 8.1 | 8.0 | 6.6, 9.6 | 84.4 | 82.0, 86.5 |
| Turkmenistan | 2016 | 0.0 | na | 0.1 | 0.0, 0.5 | 0.3 | 0.1, 0.7 | 0.6 | 0.3, 1.2 | 99.0 | 98.3, 99.4 |
| Türkiye | 2019 | 0.0 | na | 7.0 | 5.3, 9.3 | 2.4 | 1.6, 3.5 | 32.3 | 29.0, 35.7 | 58.3 | 54.5, 62.1 |
|  |  |  |  |  |  |  |  |  |  |  |  |
| Latin America & Caribbean | 2016* | 3.6 | 2.4, 5.2 | 0.3 | 0.2, 0.4 | 3.0 | 2.6, 3.5 | 17.0 | 14.8, 19.6 | 76.1 | 73.3, 78.7 |
| Belize | 2016 | 3.2 | 1.6, 6.3 | 1.0 | 0.6, 1.8 | 3.0 | 1.9, 4.7 | 19.6 | 16.1, 23.8 | 73.1 | 68.5, 77.3 |
| Costa Rica | 2018 | 0.2 | 0.0, 1.0 | 0.6 | 0.3, 1.4 | 1.8 | 1.0, 3.1 | 7.2 | 5.3, 9.7 | 90.3 | 87.5, 92.5 |
| Cuba | 2019 | 1.0 | 0.5, 1.9 | 1.1 | 0.4, 2.9 | 1.0 | 0.5, 2.0 | 11.0 | 8.1, 14.6 | 85.9 | 82.0, 89.0 |
| Dominican Republic | 2019 | 0.0 | na | 0.0 | na | 10.0 | 8.7, 11.5 | 19.4 | 17.3, 21.6 | 70.7 | 68.3, 72.9 |
| El Salvador | 2014 | 0.4 | 0.1, 0.9 | 0.3 | 0.1, 0.9 | 0.4 | 0.2, 0.9 | 10.8 | 9.3, 12.5 | 88.1 | 86.3, 89.7 |
| Guatemala | 2015 | 0.0 | na | 0.1 | 0.0, 0.3 | 2.0 | 1.5, 2.5 | 9.7 | 8.5, 10.9 | 88.3 | 86.9, 89.6 |
| Guyana | 2014 | 2.3 | 1.4, 3.5 | 0.4 | 0.2, 1.2 | 0.9 | 0.5, 1.6 | 5.3 | 3.9, 7.1 | 91.2 | 89.0, 92.9 |
| Haiti | 2017 | 0.0 | na | 0.2 | 0.1, 0.5 | 18.2 | 15.7, 21.1 | 22.8 | 20.7, 25.1 | 58.8 | 55.3, 62.1 |
| Honduras | 2019 | 1.9 | 1.4, 2.6 | 0.9 | 0.5, 1.6 | 0.6 | 0.3, 1.0 | 11.0 | 9.7, 12.4 | 85.6 | 84.0, 87.1 |
| Mexico | 2015 | 5.5 | 3.6, 8.4 | 0.2 | 0.1, 0.5 | 1.4 | 1.0, 2.1 | 19.6 | 15.9, 24.0 | 73.2 | 68.4, 77.6 |
| Paraguay | 2016 | 3.2 | 2.4, 4.2 | 0.6 | 0.3, 1.3 | 0.5 | 0.3, 1.0 | 9.3 | 7.3, 11.8 | 86.3 | 83.8, 88.5 |
| Suriname | 2018 | 11.2 | 9.0, 13.9 | 3.7 | 2.6, 5.1 | 7.9 | 5.9, 10.4 | 8.9 | 7.1, 11.1 | 68.3 | 64.5, 71.9 |
|  |  |  |  |  |  |  |  |  |  |  |  |
| Middle East & North Africa | 2016* | 1.2 | 1.0, 1.4 | 0.6 | 0.5, 0.8 | 3.1 | 2.7, 3.4 | 32.2 | 31.0, 33.4 | 63.0 | 61.8, 64.2 |
| Algeria | 2019 | 2.2 | 1.7, 2.9 | 1.1 | 0.8, 1.5 | 1.9 | 1.5, 2.4 | 11.5 | 10.3, 12.9 | 83.3 | 81.7, 84.7 |
| Egypt | 2014 | 0.0 | na | 0.0 | na | 0.7 | 0.5, 1.0 | 50.3 | 48.6, 52.0 | 49.1 | 47.3, 50.8 |
| Iraq | 2018 | 3.0 | 2.4, 3.7 | 1.4 | 1.0, 2.0 | 8.7 | 7.5, 10.0 | 18.8 | 16.9, 20.9 | 68.1 | 65.4, 70.7 |
| Jordan | 2018 | 0.0 | na | 0.2 | 0.1, 0.4 | 7.7 | 6.2, 9.6 | 22.0 | 19.6, 24.7 | 70.1 | 67.2, 72.9 |
| State of Palestine | 2020 | 0.3 | 0.1, 0.7 | 1.8 | 1.2, 2.5 | 2.6 | 1.9, 3.6 | 10.6 | 9.3, 12.2 | 84.7 | 82.7, 86.6 |
| Tunisia | 2018 | 1.9 | 1.2, 2.9 | 1.3 | 0.8, 2.1 | 1.3 | 0.8, 2.2 | 13.8 | 11.7, 16.1 | 81.7 | 79.1, 84.0 |
|  |  |  |  |  |  |  |  |  |  |  |  |
| South Asia | 2020* | 0.0 | 0.0, 0.0 | 0.3 | 0.3, 0.4 | 8.3 | 7.7, 8.9 | 18.6 | 18.0, 19.2 | 72.8 | 71.9, 73.7 |
| Afghanistan | 2015 | 0.2 | 0.0, 0.8 | 2.5 | 2.0, 3.2 | 29.0 | 25.1, 33.1 | 23.8 | 21.8, 25.8 | 44.5 | 41.2, 47.9 |
| Bangladesh | 2018 | 0.0 | na | 0.1 | 0.0, 0.3 | 1.4 | 0.9, 2.3 | 28.1 | 26.2, 30.1 | 70.4 | 68.3, 72.3 |
| India | 2021 | 0.0 | na | 0.2 | 0.2, 0.3 | 6.4 | 6.2, 6.7 | 13.9 | 13.5, 14.3 | 79.5 | 79.0, 79.9 |
| Maldives | 2017 | 0.0 | na | 1.0 | 0.4, 2.1 | 8.4 | 6.4, 10.9 | 12.9 | 10.2, 16.3 | 77.7 | 74.2, 80.9 |
| Nepal | 2022 | 0.0 | na | 0.5 | 0.3, 1.1 | 5.5 | 4.3, 7.0 | 24.7 | 22.1, 27.5 | 69.3 | 66.5, 71.9 |
| Pakistan | 2018 | 0.0 | na | 0.3 | 0.1, 0.5 | 15.1 | 12.2, 18.4 | 30.6 | 28.0, 33.4 | 54.1 | 50.3, 57.8 |
|  |  |  |  |  |  |  |  |  |  |  |  |
| West & Central Africa | 2019* | 0.2 | 0.2, 0.2 | 0.5 | 0.4, 0.6 | 25.7 | 24.4, 27.0 | 26.9 | 26.1, 27.7 | 46.7 | 45.6, 47.9 |
| Benin | 2018 | 0.0 | na | 0.2 | 0.1, 0.4 | 16.3 | 14.4, 18.4 | 20.9 | 19.5, 22.5 | 62.6 | 60.4, 64.7 |
| Burkina Faso | 2021 | 0.0 | na | 0.6 | 0.4, 1.0 | 5.3 | 4.2, 6.7 | 12.6 | 11.2, 14.0 | 81.5 | 79.6, 83.3 |
| Cameroon | 2019 | 0.0 | na | 0.7 | 0.4, 1.3 | 16.2 | 13.9, 18.8 | 22.8 | 20.1, 25.8 | 60.3 | 56.8, 63.7 |
| Chad | 2015 | 0.2 | 0.1, 0.4 | 1.9 | 1.4, 2.5 | 41.0 | 38.0, 44.0 | 35.6 | 33.2, 38.0 | 21.3 | 19.1, 23.8 |
| Congo | 2015 | 6.4 | 5.2, 7.9 | 1.0 | 0.7, 1.5 | 7.2 | 6.1, 8.6 | 44.9 | 42.0, 47.9 | 40.4 | 37.2, 43.6 |
| Cote d'Ivoire | 2021 | 0.0 | na | 0.8 | 0.5, 1.5 | 29.2 | 26.7, 31.9 | 15.9 | 14.1, 17.9 | 54.0 | 51.1, 57.0 |
| Gabon | 2021 | 0.0 | na | 2.1 | 1.2, 3.6 | 16.2 | 13.5, 19.4 | 19.6 | 17.1, 22.3 | 62.2 | 58.4, 65.9 |
| Gambia | 2020 | 0.0 | na | 0.1 | 0.0, 0.4 | 2.0 | 1.4, 2.8 | 10.3 | 8.9, 11.8 | 87.6 | 85.9, 89.2 |
| Ghana | 2023 | 0.0 | na | 0.2 | 0.1, 0.5 | 3.3 | 2.5, 4.3 | 12.8 | 11.3, 14.5 | 83.7 | 81.8, 85.4 |
| Guinea | 2018 | 0.0 | na | 0.9 | 0.6, 1.5 | 35.9 | 32.4, 39.4 | 20.0 | 17.6, 22.7 | 43.2 | 39.8, 46.7 |
| Guinea-Bissau | 2019 | 6.1 | 4.9, 7.5 | 0.6 | 0.3, 1.0 | 1.9 | 1.3, 2.7 | 14.5 | 12.8, 16.4 | 77.0 | 74.7, 79.1 |
| Liberia | 2020 | 0.0 | na | 0.3 | 0.1, 1.1 | 9.3 | 7.4, 11.7 | 33.7 | 30.7, 36.8 | 56.6 | 53.2, 60.0 |
| Mali | 2018 | 0.0 | na | 0.8 | 0.5, 1.3 | 17.3 | 14.9, 19.9 | 34.2 | 31.8, 36.6 | 47.7 | 44.7, 50.8 |
| Mauritania | 2021 | 0.0 | na | 0.8 | 0.6, 1.2 | 11.9 | 10.4, 13.6 | 61.6 | 59.4, 63.8 | 25.6 | 23.8, 27.5 |
| Nigeria | 2018 | 0.0 | na | 0.3 | 0.2, 0.5 | 36.0 | 33.8, 38.2 | 30.9 | 29.5, 32.4 | 32.8 | 31.1, 34.5 |
| Sao Tome and Principe | 2019 | 1.2 | 0.7, 2.3 | 1.4 | 0.7, 2.7 | 1.6 | 0.9, 2.8 | 6.4 | 4.5, 9.1 | 89.3 | 86.5, 91.6 |
| Senegal | 2019 | 0.0 | na | 0.0 | na | 4.1 | 2.9, 5.7 | 23.2 | 20.7, 26.0 | 72.7 | 69.7, 75.5 |
| Sierra Leone | 2019 | 0.0 | na | 0.9 | 0.6, 1.4 | 4.9 | 4.0, 6.0 | 26.6 | 24.5, 28.8 | 67.6 | 65.4, 69.8 |
| Togo | 2017 | 4.0 | 3.0, 5.3 | 0.8 | 0.4, 1.5 | 5.6 | 4.4, 7.1 | 23.9 | 21.7, 26.4 | 65.7 | 62.8, 68.6 |

Notes: Estimates refer to the latest survey in each country. 95% confidence intervals (CI) were adjusted for clustering at the level of primary sampling units. Estimates were weighted using sampling weights rescaled to sum up the population 12–35 months old in the country and year of survey.

# Sensitivity analysis: Excluding children with missing DPT vaccine information (instead of coding them as zero-dose)

## Figure S1. Distribution of zero-dose prevalence across countries by survey year: excluding children with missing DPT vaccine information (instead of coding them as zero-dose)

Note: Only includes countries with two surveys. Percentiles 5 and 95 (line) and 25, 50, and 75 (box) are shown. Dots indicate country estimates. Each country's estimate was weighted using sampling weights. Surveys were equally weighted for the median and percentiles. See Tables 2 and S2 for tabulated estimates. Percentage point (pp) average annual change (AAC) is shown on the right side y-axis.

## Table S4. Tabulated estimates from Figure 1: Distribution of zero-dose prevalence across countries: excluding children with missing DPT vaccine information (instead of coding them as zero-dose)

|  | Earlier surveys (%) | Later surveys (%) | AAC (pp) |
| --- | --- | --- | --- |
|  |  |  |  |
| Percentile 5 | 0.8 | 1.4 | -1.480 |
| Percentile 25 | 3.5 | 2.8 | -0.770 |
| Median | 9.9 | 6.3 | -0.218 |
| Percentile 75 | 23.4 | 13.0 | 0.153 |
| Percentile 95 | 45.7 | 32.2 | 0.888 |
| Interquartile range | 19.9 | 10.3 | 0.924 |
| Mean | 14.5 | 9.8 | -0.287 |

Notes: Only countries with two surveys were included. Each country's estimate was weighted using sampling weights. Surveys were equally weighted for the median and percentiles. Percentage point (pp) average annual change (AAC) is shown.

## Table S5. Zero-dose prevalence and estimated number of zero-dose children: excluding children with missing DPT vaccine information (instead of coding them as zero-dose)

|  | Survey | Prevalence | 95% confidence | Number |
| --- | --- | --- | --- | --- |
|  | year | (%) | interval | (thousands) |
|  |  |  |  |  |
| Pooled | 2019* | 12.0 | 11.5, 12.4 | 20,356 |
|  |  |  |  |  |
| East Asia & Pacific | 2019* | 11.0 | 10.2, 11.8 | 2,256 |
| Cambodia | 2022 | 7.3 | 6.1, 8.6 | 46 |
| Fiji | 2021 | 1.2 | 0.6, 2.2 | 0.41 |
| Indonesia | 2017 | 10.5 | 9.4, 11.8 | 984 |
| Lao | 2017 | 18.9 | 17.1, 20.8 | 59 |
| Mongolia | 2018 | 2.2 | 1.3, 3.5 | 3.4 |
| Myanmar | 2016 | 13.0 | 10.4, 16.2 | 234 |
| Papua New Guinea | 2018 | 36.0 | 32.7, 39.4 | 170 |
| Philippines | 2022 | 13.0 | 11.0, 15.3 | 627 |
| Samoa | 2020 | 13.4 | 10.5, 16.9 | 1.6 |
| Timor-Leste | 2016 | 22.8 | 20.2, 25.6 | 14 |
| Tonga | 2019 | 4.6 | 2.7, 7.7 | 0.23 |
| Tuvalu | 2020 | 2.0 | 0.8, 5.0 | 0.010 |
| Viet Nam | 2021 | 4.3 | 3.1, 6.0 | 128 |
|  |  |  |  |  |
| Eastern & Southern Africa | 2018* | 13.7 | 12.3, 15.1 | 4,409 |
| Angola | 2016 | 32.2 | 29.2, 35.3 | 692 |
| Burundi | 2017 | 0.9 | 0.7, 1.3 | 7.8 |
| Eswatini | 2014 | 2.0 | 1.3, 3.0 | 1.2 |
| Ethiopia | 2019 | 29.9 | 25.0, 35.3 | 2,051 |
| Kenya | 2022 | 3.5 | 2.9, 4.2 | 97 |
| Lesotho | 2014 | 2.1 | 1.4, 3.1 | 2.3 |
| Madagascar | 2021 | 22.6 | 20.2, 25.1 | 378 |
| Malawi | 2016 | 2.8 | 2.2, 3.4 | 32 |
| Mozambique | 2015 | 11.7 | 9.0, 14.9 | 220 |
| Rwanda | 2020 | 0.5 | 0.3, 0.8 | 3.6 |
| South Africa | 2016 | 10.5 | 8.4, 13.2 | 245 |
| Sudan | 2014 | 6.1 | 4.9, 7.5 | 147 |
| Tanzania | 2022 | 5.2 | 4.3, 6.3 | 227 |
| Uganda | 2016 | 5.9 | 5.1, 6.8 | 165 |
| Zambia | 2019 | 2.3 | 1.7, 3.1 | 27 |
| Zimbabwe | 2015 | 12.0 | 10.0, 14.4 | 111 |
|  |  |  |  |  |
| Europe & Central Asia | 2018* | 7.1 | 5.9, 8.4 | 352 |
| Armenia | 2016 | 2.6 | 1.6, 4.4 | 2.3 |
| Kazakhstan | 2015 | 2.6 | 1.9, 3.6 | 20 |
| Kosovo | 2020 | 3.4 | 2.1, 5.3 | 1.3 |
| Kyrgyzstan | 2018 | 7.1 | 5.5, 9.2 | 23 |
| North Macedonia | 2019 | 2.3 | 1.0, 5.4 | 1.0 |
| Serbia | 2019 | 3.3 | 2.0, 5.5 | 4.6 |
| Tajikistan | 2017 | 7.4 | 6.0, 9.2 | 38 |
| Turkmenistan | 2016 | 0.4 | 0.2, 0.9 | 1.2 |
| Türkiye | 2019 | 9.4 | 7.4, 11.8 | 262 |
|  |  |  |  |  |
| Latin America & Caribbean | 2016* | 3.3 | 2.9, 3.9 | 247 |
| Belize | 2016 | 4.1 | 2.8, 6.2 | 0.64 |
| Costa Rica | 2018 | 2.4 | 1.5, 3.8 | 3.3 |
| Cuba | 2019 | 2.2 | 1.2, 3.9 | 5.0 |
| Dominican Republic | 2019 | 10.0 | 8.7, 11.5 | 41 |
| El Salvador | 2014 | 0.7 | 0.4, 1.4 | 1.7 |
| Guatemala | 2015 | 2.0 | 1.6, 2.6 | 16 |
| Guyana | 2014 | 1.4 | 0.7, 2.4 | 0.42 |
| Haiti | 2017 | 18.4 | 15.8, 21.3 | 93 |
| Honduras | 2019 | 1.5 | 1.0, 2.3 | 6.4 |
| Mexico | 2015 | 1.7 | 1.2, 2.5 | 75 |
| Paraguay | 2016 | 1.2 | 0.8, 1.9 | 3.2 |
| Suriname | 2018 | 13.0 | 10.4, 16.2 | 2.8 |
|  |  |  |  |  |
| Middle East & North Africa | 2016* | 3.8 | 3.4, 4.2 | 397 |
| Algeria | 2019 | 3.0 | 2.5, 3.7 | 60 |
| Egypt | 2014 | 0.8 | 0.5, 1.2 | 40 |
| Iraq | 2018 | 10.4 | 9.0, 11.9 | 236 |
| Jordan | 2018 | 7.8 | 6.3, 9.7 | 37 |
| State of Palestine | 2020 | 4.4 | 3.4, 5.6 | 12 |
| Tunisia | 2018 | 2.7 | 1.9, 3.8 | 12 |
|  |  |  |  |  |
| South Asia | 2020* | 8.5 | 8.0, 9.2 | 5,714 |
| Afghanistan | 2015 | 31.6 | 27.7, 35.7 | 738 |
| Bangladesh | 2018 | 1.5 | 1.0, 2.3 | 87 |
| India | 2021 | 6.7 | 6.4, 7.0 | 3,056 |
| Maldives | 2017 | 9.3 | 7.0, 12.3 | 1.4 |
| Nepal | 2022 | 6.1 | 4.8, 7.6 | 72 |
| Pakistan | 2018 | 15.3 | 12.5, 18.7 | 1,789 |
|  |  |  |  |  |
| West & Central Africa | 2019* | 25.9 | 24.6, 27.2 | 7,137 |
| Benin | 2018 | 16.5 | 14.6, 18.6 | 134 |
| Burkina Faso | 2021 | 5.9 | 4.8, 7.3 | 85 |
| Cameroon | 2019 | 16.9 | 14.5, 19.5 | 288 |
| Chad | 2015 | 43.0 | 40.0, 46.0 | 487 |
| Congo | 2015 | 8.8 | 7.5, 10.3 | 30 |
| Cote d'Ivoire | 2021 | 30.0 | 27.5, 32.7 | 507 |
| Gabon | 2021 | 16.1 | 13.7, 18.8 | 20 |
| Gambia | 2020 | 2.1 | 1.5, 2.9 | 3.5 |
| Ghana | 2023 | 3.5 | 2.7, 4.5 | 62 |
| Guinea | 2018 | 36.8 | 33.3, 40.4 | 301 |
| Guinea-Bissau | 2019 | 2.6 | 1.9, 3.5 | 3.1 |
| Liberia | 2020 | 9.7 | 7.7, 12.1 | 29 |
| Mali | 2018 | 18.1 | 15.6, 20.8 | 273 |
| Mauritania | 2021 | 12.8 | 11.2, 14.5 | 36 |
| Nigeria | 2018 | 36.3 | 34.1, 38.5 | 4,864 |
| Sao Tome and Principe | 2019 | 3.1 | 2.0, 4.6 | 0.37 |
| Senegal | 2019 | 4.1 | 2.9, 5.7 | 41 |
| Sierra Leone | 2019 | 5.8 | 4.8, 7.0 | 27 |
| Togo | 2017 | 6.6 | 5.2, 8.2 | 32 |

Note: *Average survey year is shown for pooled and regional estimates. The number of zero-dose children was estimated using the zero-dose prevalence and an estimate of the population of children 12–35 months old obtained from the United Nation World Population Prospects linked to the country and year of survey. Estimates refer to the latest survey in each country. 95% confidence intervals (CI) were adjusted for clustering at the level of primary sampling units. Estimates were weighted using sampling weights rescaled to sum up the population 12–35 months old in the country and year of survey.

## Table S6. Zero-dose prevalence and average annual percentage point (pp) change in prevalence: excluding children with missing DPT vaccine information (instead of coding them as zero-dose)

|  | Earlier Survey | | | Later Survey | | | Average annual change | |
| --- | --- | --- | --- | --- | --- | --- | --- | --- |
|  | Survey | Zero-dose | 95% CI | Survey | Zero-dose | 95% CI | Change | 95% CI |
|  | year | % | % | year | % | % | pp | % |
|  |  |  |  |  |  |  |  |  |
| Pooled | 2005** | 22.4 | 21.6, 23.2 | 2019** | 12.0 | 11.6, 12.5 | -0.7* | -0.8, -0.7 |
|  |  |  |  |  |  |  |  |  |
| East Asia & Pacific | 2002** | 15.7 | 14.3, 17.2 | 2019** | 10.4 | 9.6, 11.3 | -0.3* | -0.4, -0.2 |
| Cambodia | 2000 | 34.0 | 30.8, 37.4 | 2022 | 7.3 | 6.1, 8.6 | -1.2* | -1.4, -1.1 |
| Indonesia | 2003 | 19.5 | 17.0, 22.3 | 2017 | 10.5 | 9.4, 11.8 | -0.6* | -0.8, -0.4 |
| Lao | 2000 | 36.6 | 32.6, 40.7 | 2017 | 18.9 | 17.1, 20.8 | -1.0* | -1.3, -0.8 |
| Mongolia | 2000 | 6.4 | 4.8, 8.5 | 2018 | 2.2 | 1.3, 3.5 | -0.2* | -0.4, -0.1 |
| Myanmar | 2000 | 10.1 | 8.8, 11.6 | 2016 | 13.0 | 10.4, 16.2 | 0.2 | -0.0, 0.4 |
| Philippines | 2003 | 10.4 | 9.0, 12.0 | 2022 | 13.0 | 11.0, 15.3 | 0.1 | -0.0, 0.3 |
| Timor-Leste | 2010 | 25.6 | 23.0, 28.3 | 2016 | 22.8 | 20.2, 25.6 | -0.5 | -1.1, 0.2 |
| Viet Nam | 2002 | 9.8 | 6.7, 14.1 | 2021 | 4.3 | 3.1, 6.0 | -0.3* | -0.5, -0.1 |
|  |  |  |  |  |  |  |  |  |
| Eastern & Southern Africa | 2003** | 24.9 | 23.4, 26.3 | 2018** | 13.9 | 12.5, 15.5 | -0.7* | -0.9, -0.6 |
| Angola | 2001 | 45.7 | 42.6, 48.8 | 2016 | 32.2 | 29.2, 35.3 | -0.9* | -1.2, -0.6 |
| Burundi | 2011 | 1.1 | 0.7, 1.5 | 2017 | 0.9 | 0.7, 1.3 | -0.0 | -0.1, 0.1 |
| Eswatini | 2007 | 3.2 | 2.2, 4.7 | 2014 | 2.0 | 1.3, 3.0 | -0.2 | -0.4, 0.0 |
| Ethiopia | 2000 | 53.8 | 50.3, 57.4 | 2019 | 29.9 | 25.0, 35.3 | -1.3* | -1.6, -0.9 |
| Kenya | 2009 | 5.2 | 4.0, 6.7 | 2022 | 3.5 | 2.9, 4.2 | -0.1* | -0.2, -0.0 |
| Lesotho | 2005 | 6.0 | 4.6, 7.9 | 2014 | 2.1 | 1.4, 3.1 | -0.4* | -0.6, -0.2 |
| Madagascar | 2004 | 27.4 | 21.8, 33.8 | 2021 | 22.6 | 20.2, 25.1 | -0.3 | -0.7, 0.1 |
| Malawi | 2000 | 3.7 | 3.0, 4.6 | 2016 | 2.8 | 2.2, 3.4 | -0.1 | -0.1, 0.0 |
| Mozambique | 2004 | 14.0 | 11.8, 16.7 | 2015 | 11.7 | 9.0, 14.9 | -0.2 | -0.6, 0.1 |
| Rwanda | 2000 | 5.0 | 4.1, 6.1 | 2020 | 0.5 | 0.3, 0.8 | -0.2* | -0.3, -0.2 |
| Sudan | 2000 | 32.2 | 30.2, 34.3 | 2014 | 6.1 | 4.9, 7.5 | -1.9* | -2.0, -1.7 |
| Tanzania | 2005 | 6.8 | 5.2, 9.0 | 2022 | 5.2 | 4.3, 6.3 | -0.1 | -0.2, 0.0 |
| Uganda | 2001 | 20.3 | 17.7, 23.2 | 2016 | 5.9 | 5.1, 6.8 | -1.0* | -1.2, -0.8 |
| Zambia | 2002 | 6.0 | 4.8, 7.4 | 2019 | 2.3 | 1.7, 3.1 | -0.2* | -0.3, -0.1 |
| Zimbabwe | 2006 | 26.8 | 23.8, 30.1 | 2015 | 12.0 | 10.0, 14.4 | -1.6* | -2.1, -1.2 |
|  |  |  |  |  |  |  |  |  |
| Europe & Central Asia | 2007** | 7.7 | 6.2, 9.4 | 2018** | 7.5 | 6.3, 9.0 | -0.0 | -0.2, 0.2 |
| Armenia | 2000 | 7.1 | 5.0, 9.9 | 2016 | 2.6 | 1.6, 4.4 | -0.3* | -0.5, -0.1 |
| Kazakhstan | 2011 | 1.2 | 0.7, 2.0 | 2015 | 2.6 | 1.9, 3.6 | 0.4* | 0.1, 0.6 |
| Kosovo | 2014 | 1.7 | 0.9, 3.3 | 2020 | 3.4 | 2.1, 5.3 | 0.3 | -0.0, 0.6 |
| Kyrgyzstan | 2012 | 1.8 | 1.1, 2.9 | 2018 | 7.1 | 5.5, 9.2 | 0.9* | 0.5, 1.2 |
| Serbia | 2006 | 2.8 | 1.9, 4.1 | 2019 | 3.3 | 2.0, 5.5 | 0.0 | -0.1, 0.2 |
| Tajikistan | 2012 | 3.8 | 2.8, 5.0 | 2017 | 7.4 | 6.0, 9.2 | 0.7* | 0.3, 1.1 |
| Türkiye | 2004 | 11.3 | 9.0, 14.2 | 2019 | 9.4 | 7.4, 11.8 | -0.1 | -0.4, 0.1 |
|  |  |  |  |  |  |  |  |  |
| Latin America & Caribbean | 2004** | 7.1 | 5.9, 8.5 | 2018** | 8.4 | 7.5, 9.4 | 0.1 | -0.0, 0.2 |
| Belize | 2011 | 2.7 | 1.6, 4.6 | 2016 | 4.1 | 2.8, 6.2 | 0.3 | -0.2, 0.7 |
| Costa Rica | 2011 | 1.6 | 0.7, 3.3 | 2018 | 2.4 | 1.5, 3.8 | 0.1 | -0.1, 0.3 |
| Cuba | 2006 | 0.5 | 0.2, 0.9 | 2019 | 2.2 | 1.2, 3.9 | 0.1* | 0.0, 0.2 |
| Dominican Republic | 2002 | 5.5 | 4.6, 6.4 | 2019 | 10.0 | 8.7, 11.5 | 0.3* | 0.2, 0.4 |
| Guyana | 2009 | 8.8 | 6.7, 11.3 | 2014 | 1.4 | 0.7, 2.4 | -1.5* | -2.0, -1.0 |
| Haiti | 2000 | 20.6 | 16.7, 25.1 | 2017 | 18.4 | 15.8, 21.3 | -0.1 | -0.4, 0.2 |
| Honduras | 2006 | 0.9 | 0.6, 1.4 | 2019 | 1.5 | 1.0, 2.3 | 0.0 | -0.0, 0.1 |
| Suriname | 2001 | 10.1 | 7.7, 13.3 | 2018 | 13.0 | 10.4, 16.2 | 0.2 | -0.1, 0.4 |
|  |  |  |  |  |  |  |  |  |
| Middle East & North Africa | 2006** | 3.4 | 3.1, 3.7 | 2016** | 3.8 | 3.4, 4.2 | 0.0 | -0.0, 0.1 |
| Algeria | 2013 | 1.8 | 1.5, 2.3 | 2019 | 3.0 | 2.5, 3.7 | 0.2* | 0.1, 0.3 |
| Egypt | 2000 | 0.8 | 0.5, 1.2 | 2014 | 0.8 | 0.5, 1.2 | -0.0 | -0.0, 0.0 |
| Iraq | 2011 | 10.6 | 9.7, 11.6 | 2018 | 10.4 | 9.0, 11.9 | -0.0 | -0.3, 0.2 |
| Jordan | 2002 | 0.4 | 0.2, 0.8 | 2018 | 7.8 | 6.3, 9.7 | 0.5* | 0.4, 0.6 |
| State of Palestine | 2010 | 0.9 | 0.7, 1.3 | 2020 | 4.4 | 3.4, 5.6 | 0.3* | 0.2, 0.5 |
| Tunisia | 2013 | 0.7 | 0.3, 1.4 | 2018 | 2.7 | 1.9, 3.8 | 0.4* | 0.2, 0.6 |
|  |  |  |  |  |  |  |  |  |
| South Asia | 2006** | 23.2 | 22.1, 24.4 | 2020** | 7.7 | 7.1, 8.4 | -1.1* | -1.2, -1.0 |
| Bangladesh | 2004 | 6.9 | 5.4, 8.9 | 2018 | 1.5 | 1.0, 2.3 | -0.4* | -0.5, -0.3 |
| India | 2006 | 25.1 | 23.7, 26.6 | 2021 | 6.7 | 6.4, 7.0 | -1.2* | -1.3, -1.1 |
| Maldives | 2009 | 0.8 | 0.4, 1.5 | 2017 | 9.3 | 7.0, 12.3 | 1.1* | 0.7, 1.4 |
| Nepal | 2001 | 15.2 | 12.1, 18.9 | 2022 | 6.1 | 4.8, 7.6 | -0.4* | -0.6, -0.3 |
| Pakistan | 2007 | 25.7 | 23.4, 28.2 | 2018 | 15.3 | 12.5, 18.7 | -0.9* | -1.3, -0.6 |
|  |  |  |  |  |  |  |  |  |
| West & Central Africa | 2004** | 37.0 | 34.0, 40.0 | 2019** | 25.9 | 24.6, 27.2 | -0.8* | -1.0, -0.5 |
| Benin | 2001 | 12.8 | 10.4, 15.7 | 2018 | 16.5 | 14.6, 18.6 | 0.2* | 0.0, 0.4 |
| Burkina Faso | 2003 | 23.1 | 19.8, 26.8 | 2021 | 5.9 | 4.8, 7.3 | -1.0* | -1.2, -0.7 |
| Cameroon | 2004 | 17.1 | 14.7, 19.7 | 2019 | 16.9 | 14.5, 19.5 | -0.0 | -0.2, 0.2 |
| Chad | 2004 | 56.4 | 50.6, 62.1 | 2015 | 43.0 | 40.0, 46.0 | -1.2* | -1.8, -0.6 |
| Congo | 2005 | 14.2 | 11.4, 17.5 | 2015 | 8.8 | 7.5, 10.3 | -0.5* | -0.9, -0.2 |
| Cote d'Ivoire | 2012 | 20.6 | 17.7, 23.8 | 2021 | 30.0 | 27.5, 32.7 | 1.0* | 0.6, 1.5 |
| Gabon | 2001 | 26.5 | 23.6, 29.5 | 2021 | 16.1 | 13.7, 18.8 | -0.5* | -0.7, -0.3 |
| Gambia | 2013 | 2.6 | 1.9, 3.6 | 2020 | 2.1 | 1.5, 2.9 | -0.1 | -0.2, 0.1 |
| Ghana | 2003 | 9.9 | 8.0, 12.3 | 2023 | 3.5 | 2.7, 4.5 | -0.3* | -0.4, -0.2 |
| Guinea | 2005 | 22.7 | 19.7, 25.9 | 2018 | 36.8 | 33.3, 40.4 | 1.1* | 0.7, 1.4 |
| Guinea-Bissau | 2000 | 26.5 | 26.5, 26.5 | 2019 | 2.6 | 1.9, 3.5 | -1.3* | -1.3, -1.2 |
| Liberia | 2007 | 24.6 | 19.7, 30.4 | 2020 | 9.7 | 7.7, 12.1 | -1.2* | -1.6, -0.7 |
| Mali | 2001 | 37.2 | 34.2, 40.3 | 2018 | 18.1 | 15.6, 20.8 | -1.1* | -1.4, -0.9 |
| Mauritania | 2011 | 8.3 | 7.1, 9.8 | 2021 | 12.8 | 11.2, 14.5 | 0.4* | 0.2, 0.7 |
| Nigeria | 2003 | 55.8 | 50.5, 60.9 | 2018 | 36.3 | 34.1, 38.5 | -1.3* | -1.7, -0.9 |
| Sao Tome and Principe | 2009 | 6.8 | 4.9, 9.4 | 2019 | 3.1 | 2.0, 4.6 | -0.4* | -0.6, -0.1 |
| Senegal | 2005 | 7.7 | 6.5, 9.0 | 2019 | 4.1 | 2.9, 5.7 | -0.3* | -0.4, -0.1 |
| Sierra Leone | 2008 | 23.7 | 20.9, 26.7 | 2019 | 5.8 | 4.8, 7.0 | -1.6* | -1.9, -1.3 |
| Togo | 2014 | 7.3 | 5.8, 9.2 | 2017 | 6.6 | 5.2, 8.2 | -0.2 | -1.0, 0.5 |

Note: *p<0.05. **Average survey year is shown for pooled and regional estimates. 95% confidence intervals (CI) were adjusted for clustering at the level of primary sampling units. Estimates were weighted using sampling weights rescaled to sum up the population 12–35 months old in the country and year of survey.

## Figure S2. The relationship between country-level zero-dose prevalence in the earliest survey and average annual percentage point (pp) change in prevalence: excluding children with missing DPT vaccine information (instead of coding them as zero-dose)

Note: Pearson's correlation coefficient (r) is shown. Countries were equally weighted.

## Figure S3. Correlation of zero-dose prevalence with health expenditure and postneonatal and child mortality rate: excluding children with missing DPT vaccine information (instead of coding them as zero-dose)

Notes: The y and x-axes vary across graphs. GDP and health expenditure were measured per capita in PPP adjusted constant 2017 international $. Pearson's correlation coefficients (r) are shown. Postneonatal and child mortality rate is deaths per 1,000 live births. ∆ indicates variables expressed as average annual (absolute) change. Countries were equally weighted.

## Table S7. Linear regressions: excluding children with missing DPT vaccine information (instead of coding them as zero-dose)

|  | (1) | (2) | (3) | (4) |
| --- | --- | --- | --- | --- |
|  | Level in latest survey | | Average annual change | |
|  | Zero-dose | Postneonatal | Zero-dose | Postneonatal |
|  | prevalence | and child | prevalence | and child |
|  |  | mortality |  | mortality |
|  |  |  |  |  |
| Zero-dose prevalence |  | 0.63* |  | 1.13* |
|  |  | (0.25) |  | (0.28) |
| Total health expenditure (ln) | -7.50* | -1.63 | 1 | -6.05 |
|  | (2.91) | (4.62) | (2.50) | (7.81) |
| GDP (ln) | 6.39* | -6.69 | 1.65 | 7.49 |
|  | (2.84) | (4.73) | (6.05) | (12.53) |
| Population below age 5 (%) | -0.24* | -0.49* | 0.06 | -0.14 |
|  | (0.09) | (0.12) | (0.03) | (0.08) |
| Survey year | 0.12 | -0.87 | 0.03 | 0.08 |
|  | (0.41) | (0.53) | (0.03) | (0.09) |
| Gavi-eligible |  |  | -0.61* |  |
|  |  |  | (0.14) |  |
| Constant | 9.80* | 25.52* | -0.29* | -2.03* |
|  | (0.98) | (1.40) | (0.08) | (0.19) |
|  |  |  |  |  |
| R squared | 0.31 | 0.67 | 0.20 | 0.24 |
| Observations | 78 | 78 | 64 | 64 |

Note: *P<0.05. Dependent variables are indicated at the top of each column. In columns 3 and 4, all variables are expressed as average annual absolute change, except for survey year and Gavi-eligibility. All values were mean-centered. GDP and health expenditure are per capita in PPP adjusted constant 2017 international $. Postneonatal and child mortality rate is expressed as deaths per 1,000 live births. Countries were equally weighted. Robust standard errors are shown in parentheses below coefficients.

# Sensitivity analysis: Excluding children with missing DPT vaccine information and whose mothers answered that they did not know whether the child was vaccinated with DPT (instead of coding them as zero-dose)

## Figure S4. Distribution of zero-dose prevalence across countries by survey year: excluding children with missing DPT vaccine information and whose mothers answered that they did not know whether the child was vaccinated with DPT (instead of coding them as zero-dose)

Note: Only includes countries with two surveys. Percentiles 5 and 95 (line) and 25, 50, and 75 (box) are shown. Dots indicate country estimates. Each country's estimate was weighted using sampling weights. Surveys were equally weighted for the median and percentiles. See Tables 2 and S2 for tabulated estimates. Percentage point (pp) average annual change (AAC) is shown on the right side y-axis.

## Table S8. Tabulated estimates from Figure 1: Distribution of zero-dose prevalence across countries: excluding children with missing DPT vaccine information and whose mothers answered that they did not know whether the child was vaccinated with DPT (instead of coding them as zero-dose)

|  | Earlier surveys (%) | Later surveys (%) | AAC (pp) |
| --- | --- | --- | --- |
|  |  |  |  |
| Percentile 5 | 0.5 | 0.9 | -1.413 |
| Percentile 25 | 2.9 | 2.0 | -0.714 |
| Median | 8.7 | 5.5 | -0.242 |
| Percentile 75 | 22.6 | 12.3 | 0.070 |
| Percentile 95 | 45.7 | 30.8 | 0.937 |
| Interquartile range | 19.6 | 10.3 | 0.784 |
| Mean | 14.0 | 9.0 | -0.317 |

Notes: Only countries with two surveys were included. Each country's estimate was weighted using sampling weights. Surveys were equally weighted for the median and percentiles. Percentage point (pp) average annual change (AAC) is shown.

## Table S9. Zero-dose prevalence and estimated number of zero-dose children: excluding children with missing DPT vaccine information and whose mothers answered that they did not know whether the child was vaccinated with DPT (instead of coding them as zero-dose)

|  | Survey | Prevalence | 95% confidence | Number |
| --- | --- | --- | --- | --- |
|  | year | (%) | interval | (thousands) |
|  |  |  |  |  |
| Pooled | 2019* | 11.4 | 10.9, 11.8 | 19,331 |
|  |  |  |  |  |
| East Asia & Pacific | 2019* | 10.5 | 9.7, 11.4 | 2,168 |
| Cambodia | 2022 | 6.6 | 5.5, 7.9 | 42 |
| Fiji | 2021 | 0.7 | 0.3, 1.7 | 0.24 |
| Indonesia | 2017 | 10.2 | 9.0, 11.5 | 950 |
| Lao | 2017 | 16.2 | 14.6, 18.0 | 51 |
| Mongolia | 2018 | 1.9 | 1.1, 3.1 | 2.9 |
| Myanmar | 2016 | 12.6 | 10.0, 15.7 | 225 |
| Papua New Guinea | 2018 | 34.4 | 31.1, 37.8 | 163 |
| Philippines | 2022 | 12.5 | 10.5, 14.8 | 605 |
| Samoa | 2020 | 12.4 | 9.6, 15.9 | 1.5 |
| Timor-Leste | 2016 | 22.5 | 19.9, 25.3 | 14 |
| Tonga | 2019 | 4.6 | 2.7, 7.7 | 0.23 |
| Tuvalu | 2020 | 0.5 | 0.1, 3.7 | 0.003 |
| Viet Nam | 2021 | 4.3 | 3.1, 6.0 | 128 |
|  |  |  |  |  |
| Eastern & Southern Africa | 2018* | 12.5 | 11.2, 14.0 | 4,045 |
| Angola | 2016 | 30.8 | 27.8, 34.0 | 662 |
| Burundi | 2017 | 0.9 | 0.7, 1.3 | 7.6 |
| Eswatini | 2014 | 1.1 | 0.6, 2.0 | 0.69 |
| Ethiopia | 2019 | 28.8 | 24.0, 34.1 | 1,974 |
| Kenya | 2022 | 3.2 | 2.6, 3.9 | 89 |
| Lesotho | 2014 | 1.6 | 1.0, 2.5 | 1.7 |
| Madagascar | 2021 | 22.4 | 20.0, 25.0 | 375 |
| Malawi | 2016 | 2.6 | 2.1, 3.3 | 30 |
| Mozambique | 2015 | 8.5 | 6.0, 11.8 | 160 |
| Rwanda | 2020 | 0.5 | 0.3, 0.8 | 3.6 |
| South Africa | 2016 | 6.3 | 4.5, 8.6 | 146 |
| Sudan | 2014 | 4.5 | 3.6, 5.7 | 110 |
| Tanzania | 2022 | 4.8 | 3.9, 5.9 | 208 |
| Uganda | 2016 | 5.5 | 4.7, 6.4 | 152 |
| Zambia | 2019 | 1.9 | 1.3, 2.6 | 22 |
| Zimbabwe | 2015 | 12.0 | 9.9, 14.3 | 110 |
|  |  |  |  |  |
| Europe & Central Asia | 2018* | 3.1 | 2.5, 3.8 | 153 |
| Armenia | 2016 | 2.6 | 1.6, 4.4 | 2.3 |
| Kazakhstan | 2015 | 2.5 | 1.8, 3.5 | 19 |
| Kosovo | 2020 | 2.8 | 1.7, 4.6 | 1.1 |
| Kyrgyzstan | 2018 | 7.1 | 5.5, 9.2 | 23 |
| North Macedonia | 2019 | 2.3 | 1.0, 5.4 | 1.0 |
| Serbia | 2019 | 1.3 | 0.6, 2.8 | 1.8 |
| Tajikistan | 2017 | 6.3 | 5.0, 8.0 | 32 |
| Turkmenistan | 2016 | 0.3 | 0.1, 0.7 | 0.79 |
| Türkiye | 2019 | 2.5 | 1.7, 3.8 | 71 |
|  |  |  |  |  |
| Latin America & Caribbean | 2016* | 3.0 | 2.6, 3.5 | 226 |
| Belize | 2016 | 3.1 | 1.9, 5.0 | 0.48 |
| Costa Rica | 2018 | 1.8 | 1.0, 3.1 | 2.5 |
| Cuba | 2019 | 1.0 | 0.5, 2.0 | 2.4 |
| Dominican Republic | 2019 | 10.0 | 8.7, 11.5 | 41 |
| El Salvador | 2014 | 0.4 | 0.2, 0.9 | 0.92 |
| Guatemala | 2015 | 2.0 | 1.5, 2.5 | 15 |
| Guyana | 2014 | 0.9 | 0.5, 1.6 | 0.28 |
| Haiti | 2017 | 18.2 | 15.7, 21.1 | 92 |
| Honduras | 2019 | 0.6 | 0.3, 1.0 | 2.5 |
| Mexico | 2015 | 1.5 | 1.0, 2.2 | 66 |
| Paraguay | 2016 | 0.5 | 0.3, 1.0 | 1.4 |
| Suriname | 2018 | 9.3 | 7.0, 12.2 | 2.0 |
|  |  |  |  |  |
| Middle East & North Africa | 2016* | 3.2 | 2.8, 3.6 | 333 |
| Algeria | 2019 | 2.0 | 1.6, 2.5 | 39 |
| Egypt | 2014 | 0.8 | 0.5, 1.2 | 40 |
| Iraq | 2018 | 9.1 | 7.9, 10.5 | 207 |
| Jordan | 2018 | 7.7 | 6.2, 9.6 | 36 |
| State of Palestine | 2020 | 2.7 | 1.9, 3.7 | 7.6 |
| Tunisia | 2018 | 1.4 | 0.8, 2.3 | 6.0 |
|  |  |  |  |  |
| South Asia | 2020* | 8.3 | 7.7, 8.9 | 5,528 |
| Afghanistan | 2015 | 29.8 | 25.9, 34.0 | 697 |
| Bangladesh | 2018 | 1.4 | 0.9, 2.3 | 84 |
| India | 2021 | 6.5 | 6.2, 6.7 | 2,958 |
| Maldives | 2017 | 8.4 | 6.4, 11.0 | 1.3 |
| Nepal | 2022 | 5.6 | 4.4, 7.1 | 66 |
| Pakistan | 2018 | 15.1 | 12.3, 18.5 | 1,764 |
|  |  |  |  |  |
| West & Central Africa | 2019* | 25.5 | 24.3, 26.8 | 7,033 |
| Benin | 2018 | 16.3 | 14.4, 18.4 | 133 |
| Burkina Faso | 2021 | 5.3 | 4.2, 6.7 | 77 |
| Cameroon | 2019 | 16.3 | 14.0, 19.0 | 278 |
| Chad | 2015 | 41.9 | 38.9, 45.0 | 474 |
| Congo | 2015 | 7.8 | 6.6, 9.3 | 26 |
| Cote d'Ivoire | 2021 | 29.4 | 26.9, 32.1 | 497 |
| Gabon | 2021 | 14.3 | 12.1, 16.9 | 18 |
| Gambia | 2020 | 2.0 | 1.4, 2.8 | 3.3 |
| Ghana | 2023 | 3.3 | 2.5, 4.3 | 59 |
| Guinea | 2018 | 36.2 | 32.7, 39.8 | 296 |
| Guinea-Bissau | 2019 | 2.0 | 1.4, 2.9 | 2.4 |
| Liberia | 2020 | 9.4 | 7.4, 11.8 | 28 |
| Mali | 2018 | 17.4 | 15.0, 20.1 | 263 |
| Mauritania | 2021 | 12.0 | 10.5, 13.8 | 34 |
| Nigeria | 2018 | 36.1 | 33.9, 38.4 | 4,838 |
| Sao Tome and Principe | 2019 | 1.7 | 1.0, 2.9 | 0.20 |
| Senegal | 2019 | 4.1 | 2.9, 5.7 | 41 |
| Sierra Leone | 2019 | 4.9 | 4.0, 6.0 | 23 |
| Togo | 2017 | 5.8 | 4.6, 7.5 | 28 |

Note: *Average survey year is shown for pooled and regional estimates. The number of zero-dose children was estimated using the zero-dose prevalence and an estimate of the population of children 12–35 months old obtained from the United Nation World Population Prospects linked to the country and year of survey. Estimates refer to the latest survey in each country. 95% confidence intervals (CI) were adjusted for clustering at the level of primary sampling units. Estimates were weighted using sampling weights rescaled to sum up the population 12–35 months old in the country and year of survey.

## Table S10. Zero-dose prevalence and average annual percentage point (pp) change in prevalence: excluding children with missing DPT vaccine information and whose mothers answered that they did not know whether the child was vaccinated with DPT (instead of coding them as zero-dose)

|  | Earlier Survey | | | Later Survey | | | Average annual change | |
| --- | --- | --- | --- | --- | --- | --- | --- | --- |
|  | Survey | Zero-dose | 95% CI | Survey | Zero-dose | 95% CI | Change | 95% CI |
|  | year | % | % | year | % | % | pp | % |
|  |  |  |  |  |  |  |  |  |
| Pooled | 2005** | 21.8 | 21.0, 22.6 | 2019** | 11.5 | 11.0, 12.0 | -0.7* | -0.8, -0.7 |
|  |  |  |  |  |  |  |  |  |
| East Asia & Pacific | 2002** | 15.5 | 14.1, 16.9 | 2019** | 10.0 | 9.2, 10.9 | -0.3* | -0.4, -0.2 |
| Cambodia | 2000 | 33.5 | 30.3, 36.9 | 2022 | 6.6 | 5.5, 7.9 | -1.2* | -1.4, -1.1 |
| Indonesia | 2003 | 19.1 | 16.7, 21.9 | 2017 | 10.2 | 9.0, 11.5 | -0.6* | -0.8, -0.4 |
| Lao | 2000 | 36.6 | 32.6, 40.7 | 2017 | 16.2 | 14.6, 18.0 | -1.2* | -1.5, -0.9 |
| Mongolia | 2000 | 6.4 | 4.8, 8.5 | 2018 | 1.9 | 1.1, 3.1 | -0.3* | -0.4, -0.1 |
| Myanmar | 2000 | 10.1 | 8.8, 11.6 | 2016 | 12.6 | 10.0, 15.7 | 0.2 | -0.0, 0.4 |
| Philippines | 2003 | 10.3 | 8.9, 11.9 | 2022 | 12.5 | 10.5, 14.8 | 0.1 | -0.0, 0.3 |
| Timor-Leste | 2010 | 25.6 | 23.0, 28.3 | 2016 | 22.5 | 19.9, 25.3 | -0.5 | -1.1, 0.1 |
| Viet Nam | 2002 | 9.7 | 6.6, 14.0 | 2021 | 4.3 | 3.1, 6.0 | -0.3* | -0.5, -0.1 |
|  |  |  |  |  |  |  |  |  |
| Eastern & Southern Africa | 2003** | 24.6 | 23.2, 26.1 | 2018** | 13.0 | 11.6, 14.5 | -0.8* | -0.9, -0.6 |
| Angola | 2001 | 45.7 | 42.6, 48.8 | 2016 | 30.8 | 27.8, 34.0 | -1.0* | -1.3, -0.7 |
| Burundi | 2011 | 1.0 | 0.7, 1.5 | 2017 | 0.9 | 0.7, 1.3 | -0.0 | -0.1, 0.1 |
| Eswatini | 2007 | 2.8 | 1.9, 4.3 | 2014 | 1.1 | 0.6, 2.0 | -0.2* | -0.4, -0.1 |
| Ethiopia | 2000 | 53.5 | 49.9, 57.0 | 2019 | 28.8 | 24.0, 34.1 | -1.3* | -1.6, -1.0 |
| Kenya | 2009 | 5.2 | 4.0, 6.7 | 2022 | 3.2 | 2.6, 3.9 | -0.2* | -0.3, -0.0 |
| Lesotho | 2005 | 5.4 | 4.1, 7.2 | 2014 | 1.6 | 1.0, 2.5 | -0.4* | -0.6, -0.2 |
| Madagascar | 2004 | 27.1 | 21.5, 33.6 | 2021 | 22.4 | 20.0, 25.0 | -0.3 | -0.7, 0.1 |
| Malawi | 2000 | 3.6 | 2.9, 4.5 | 2016 | 2.6 | 2.1, 3.3 | -0.1* | -0.1, -0.0 |
| Mozambique | 2004 | 13.9 | 11.6, 16.5 | 2015 | 8.5 | 6.0, 11.8 | -0.5* | -0.8, -0.1 |
| Rwanda | 2000 | 4.1 | 3.3, 5.0 | 2020 | 0.5 | 0.3, 0.8 | -0.2* | -0.2, -0.1 |
| Sudan | 2000 | 32.2 | 30.2, 34.3 | 2014 | 4.5 | 3.6, 5.7 | -2.0* | -2.1, -1.8 |
| Tanzania | 2005 | 6.8 | 5.2, 9.0 | 2022 | 4.8 | 3.9, 5.9 | -0.1 | -0.2, 0.0 |
| Uganda | 2001 | 20.0 | 17.4, 22.9 | 2016 | 5.5 | 4.7, 6.4 | -1.0* | -1.2, -0.8 |
| Zambia | 2002 | 5.9 | 4.8, 7.3 | 2019 | 1.9 | 1.3, 2.6 | -0.2* | -0.3, -0.2 |
| Zimbabwe | 2006 | 26.7 | 23.6, 30.0 | 2015 | 12.0 | 9.9, 14.3 | -1.6* | -2.1, -1.2 |
|  |  |  |  |  |  |  |  |  |
| Europe & Central Asia | 2007** | 5.4 | 4.3, 6.8 | 2018** | 3.3 | 2.7, 4.0 | -0.2* | -0.3, -0.1 |
| Armenia | 2000 | 7.1 | 5.0, 9.9 | 2016 | 2.6 | 1.6, 4.4 | -0.3* | -0.5, -0.1 |
| Kazakhstan | 2011 | 0.9 | 0.5, 1.7 | 2015 | 2.5 | 1.8, 3.5 | 0.4* | 0.1, 0.6 |
| Kosovo | 2014 | 1.4 | 0.7, 2.9 | 2020 | 2.8 | 1.7, 4.6 | 0.2 | -0.1, 0.5 |
| Kyrgyzstan | 2012 | 1.5 | 0.9, 2.5 | 2018 | 7.1 | 5.5, 9.2 | 0.9* | 0.6, 1.3 |
| Serbia | 2006 | 0.9 | 0.5, 1.7 | 2019 | 1.3 | 0.6, 2.8 | 0.0 | -0.1, 0.1 |
| Tajikistan | 2012 | 3.0 | 2.1, 4.2 | 2017 | 6.3 | 5.0, 8.0 | 0.7* | 0.3, 1.0 |
| Türkiye | 2004 | 7.9 | 6.0, 10.2 | 2019 | 2.5 | 1.7, 3.8 | -0.4* | -0.5, -0.2 |
|  |  |  |  |  |  |  |  |  |
| Latin America & Caribbean | 2004** | 6.7 | 5.6, 8.1 | 2018** | 7.9 | 7.0, 8.9 | 0.1 | -0.0, 0.2 |
| Belize | 2011 | 1.9 | 1.0, 3.3 | 2016 | 3.1 | 1.9, 5.0 | 0.2 | -0.1, 0.6 |
| Costa Rica | 2011 | 1.3 | 0.6, 3.0 | 2018 | 1.8 | 1.0, 3.1 | 0.1 | -0.1, 0.3 |
| Cuba | 2006 | 0.5 | 0.2, 0.9 | 2019 | 1.0 | 0.5, 2.0 | 0.0 | -0.0, 0.1 |
| Dominican Republic | 2002 | 5.2 | 4.4, 6.2 | 2019 | 10.0 | 8.7, 11.5 | 0.3* | 0.2, 0.4 |
| Guyana | 2009 | 8.0 | 6.0, 10.6 | 2014 | 0.9 | 0.5, 1.6 | -1.4* | -1.9, -0.9 |
| Haiti | 2000 | 19.9 | 16.1, 24.4 | 2017 | 18.2 | 15.7, 21.1 | -0.1 | -0.4, 0.2 |
| Honduras | 2006 | 0.6 | 0.4, 0.9 | 2019 | 0.6 | 0.3, 1.0 | 0.0 | -0.0, 0.0 |
| Suriname | 2001 | 10.1 | 7.7, 13.3 | 2018 | 9.3 | 7.0, 12.2 | -0.1 | -0.3, 0.2 |
|  |  |  |  |  |  |  |  |  |
| Middle East & North Africa | 2006** | 2.9 | 2.7, 3.2 | 2016** | 3.2 | 2.8, 3.6 | 0.0 | -0.0, 0.1 |
| Algeria | 2013 | 1.6 | 1.2, 2.0 | 2019 | 2.0 | 1.6, 2.5 | 0.1 | -0.0, 0.2 |
| Egypt | 2000 | 0.8 | 0.5, 1.1 | 2014 | 0.8 | 0.5, 1.2 | 0.0 | -0.0, 0.0 |
| Iraq | 2011 | 9.4 | 8.6, 10.3 | 2018 | 9.1 | 7.9, 10.5 | -0.0 | -0.3, 0.2 |
| Jordan | 2002 | 0.4 | 0.2, 0.8 | 2018 | 7.7 | 6.2, 9.6 | 0.5* | 0.4, 0.6 |
| State of Palestine | 2010 | 0.1 | 0.0, 0.4 | 2020 | 2.7 | 1.9, 3.7 | 0.3* | 0.2, 0.3 |
| Tunisia | 2013 | 0.2 | 0.0, 0.9 | 2018 | 1.4 | 0.8, 2.3 | 0.2* | 0.1, 0.4 |
|  |  |  |  |  |  |  |  |  |
| South Asia | 2006** | 22.6 | 21.4, 23.8 | 2020** | 7.5 | 6.9, 8.2 | -1.1* | -1.1, -1.0 |
| Bangladesh | 2004 | 6.9 | 5.4, 8.9 | 2018 | 1.4 | 0.9, 2.3 | -0.4* | -0.5, -0.3 |
| India | 2006 | 24.7 | 23.2, 26.2 | 2021 | 6.5 | 6.2, 6.7 | -1.2* | -1.3, -1.1 |
| Maldives | 2009 | 0.6 | 0.3, 1.3 | 2017 | 8.4 | 6.4, 11.0 | 1.0* | 0.7, 1.3 |
| Nepal | 2001 | 15.1 | 12.0, 18.9 | 2022 | 5.6 | 4.4, 7.1 | -0.5* | -0.6, -0.3 |
| Pakistan | 2007 | 23.8 | 21.5, 26.2 | 2018 | 15.1 | 12.3, 18.5 | -0.8* | -1.1, -0.4 |
|  |  |  |  |  |  |  |  |  |
| West & Central Africa | 2004** | 36.3 | 33.4, 39.4 | 2019** | 25.5 | 24.3, 26.8 | -0.8* | -1.0, -0.5 |
| Benin | 2001 | 12.4 | 10.0, 15.2 | 2018 | 16.3 | 14.4, 18.4 | 0.2* | 0.0, 0.4 |
| Burkina Faso | 2003 | 22.5 | 19.2, 26.2 | 2021 | 5.3 | 4.2, 6.7 | -1.0* | -1.2, -0.8 |
| Cameroon | 2004 | 16.1 | 13.9, 18.7 | 2019 | 16.3 | 14.0, 19.0 | 0.0 | -0.2, 0.2 |
| Chad | 2004 | 56.1 | 50.2, 61.8 | 2015 | 41.9 | 38.9, 45.0 | -1.3* | -1.9, -0.7 |
| Congo | 2005 | 13.5 | 10.8, 16.7 | 2015 | 7.8 | 6.6, 9.3 | -0.6* | -0.9, -0.2 |
| Cote d'Ivoire | 2012 | 20.0 | 17.1, 23.2 | 2021 | 29.4 | 26.9, 32.1 | 1.0* | 0.6, 1.5 |
| Gabon | 2001 | 25.6 | 22.8, 28.7 | 2021 | 14.3 | 12.1, 16.9 | -0.6* | -0.8, -0.4 |
| Gambia | 2013 | 2.4 | 1.8, 3.3 | 2020 | 2.0 | 1.4, 2.8 | -0.1 | -0.2, 0.1 |
| Ghana | 2003 | 9.5 | 7.6, 11.8 | 2023 | 3.3 | 2.5, 4.3 | -0.3* | -0.4, -0.2 |
| Guinea | 2005 | 22.1 | 19.2, 25.4 | 2018 | 36.2 | 32.7, 39.8 | 1.1* | 0.7, 1.4 |
| Guinea-Bissau | 2000 | 26.5 | 26.5, 26.5 | 2019 | 2.0 | 1.4, 2.9 | -1.3* | -1.3, -1.2 |
| Liberia | 2007 | 24.1 | 19.1, 29.8 | 2020 | 9.4 | 7.4, 11.8 | -1.1* | -1.6, -0.7 |
| Mali | 2001 | 36.3 | 33.3, 39.4 | 2018 | 17.4 | 15.0, 20.1 | -1.1* | -1.3, -0.9 |
| Mauritania | 2011 | 7.0 | 5.8, 8.3 | 2021 | 12.0 | 10.5, 13.8 | 0.5* | 0.3, 0.7 |
| Nigeria | 2003 | 55.2 | 49.8, 60.5 | 2018 | 36.1 | 33.9, 38.4 | -1.3* | -1.7, -0.9 |
| Sao Tome and Principe | 2009 | 6.7 | 4.7, 9.3 | 2019 | 1.7 | 1.0, 2.9 | -0.5* | -0.7, -0.3 |
| Senegal | 2005 | 7.2 | 6.1, 8.5 | 2019 | 4.1 | 2.9, 5.7 | -0.2* | -0.4, -0.1 |
| Sierra Leone | 2008 | 22.6 | 19.8, 25.6 | 2019 | 4.9 | 4.0, 6.0 | -1.6* | -1.9, -1.3 |
| Togo | 2014 | 7.3 | 5.7, 9.2 | 2017 | 5.8 | 4.6, 7.5 | -0.5 | -1.2, 0.3 |

Note: *p<0.05. **Average survey year is shown for pooled and regional estimates. 95% confidence intervals (CI) were adjusted for clustering at the level of primary sampling units. Estimates were weighted using sampling weights rescaled to sum up the population 12–35 months old in the country and year of survey.

## Figure S5. The relationship between country-level zero-dose prevalence in the earliest survey and average annual percentage point (pp) change in prevalence: excluding children with missing DPT vaccine information and whose mothers answered that they did not know whether the child was vaccinated with DPT (instead of coding them as zero-dose)

Note: Pearson's correlation coefficient (r) is shown. Countries were equally weighted.

## Figure S6. Correlation of zero-dose prevalence with health expenditure and postneonatal and child mortality rate: excluding children with missing DPT vaccine information and whose mothers answered that they did not know whether the child was vaccinated with DPT (instead of coding them as zero-dose)

Notes: The y and x-axes vary across graphs. GDP and health expenditure were measured per capita in PPP adjusted constant 2017 international $. Pearson's correlation coefficients (r) are shown. Postneonatal and child mortality rate is deaths per 1,000 live births. ∆ indicates variables expressed as average annual (absolute) change. Countries were equally weighted.

## Table S11. Linear regressions: excluding children with missing DPT vaccine information and whose mothers answered that they did not know whether the child was vaccinated with DPT (instead of coding them as zero-dose)

|  | (1) | (2) | (3) | (4) |
| --- | --- | --- | --- | --- |
|  | Level in latest survey | | Average annual change | |
|  | Zero-dose | Postneonatal | Zero-dose | Postneonatal |
|  | prevalence | and child | prevalence | and child |
|  |  | mortality |  | mortality |
|  |  |  |  |  |
| Zero-dose prevalence |  | 0.64* |  | 1.10* |
|  |  | (0.25) |  | (0.28) |
| Total health expenditure (ln) | -7.64* | -1.51 | 0.66 | -5.61 |
|  | (2.80) | (4.57) | (2.56) | (7.86) |
| GDP (ln) | 6.21* | -6.60 | 1.55 | 7.17 |
|  | (2.73) | (4.66) | (6.31) | (12.63) |
| Population below age 5 (%) | -0.24* | -0.49* | 0.05 | -0.14 |
|  | (0.09) | (0.13) | (0.03) | (0.09) |
| Survey year | 0.14 | -0.88 | 0.04 | 0.08 |
|  | (0.40) | (0.54) | (0.03) | (0.09) |
| Gavi-eligible |  |  | -0.55* |  |
|  |  |  | (0.15) |  |
| Constant | 9* | 25.52* | -0.32* | -2.03* |
|  | (0.95) | (1.41) | (0.08) | (0.19) |
|  |  |  |  |  |
| R squared | 0.33 | 0.67 | 0.17 | 0.23 |
| Observations | 78 | 78 | 64 | 64 |

Note: *P<0.05. Dependent variables are indicated at the top of each column. In columns 3 and 4, all variables are expressed as average annual absolute change, except for survey year and Gavi-eligibility. All values were mean-centered. GDP and health expenditure are per capita in PPP adjusted constant 2017 international $. Postneonatal and child mortality rate is expressed as deaths per 1,000 live births. Countries were equally weighted. Robust standard errors are shown in parentheses below coefficients.
